# A novel hybrid approach for predicting and optimizing the adsorption of methyl orange and Cr(VI) removal from aqueous solutions using fungal-cross linked chitosan integrated into graphene oxide as a cost-effective adsorbent

**DOI:** 10.1186/s13065-025-01542-x

**Published:** 2025-07-03

**Authors:** Mohammed T. M. H. Hamad

**Affiliations:** Central Laboratory for Environmental Quality Monitoring, National Water Research Center, Kalubiya, Egypt

**Keywords:** MO, Cr(VI), Adsorption, Kinetic models, ANN, RSM

## Abstract

The release of organic dyes and heavy metal ions in wastewater from various industries has become a significant environmental issue, prompting the need for effective solutions like remediation technology to reduce these pollutants in water. In this research, we fabricated a GO@Cs-GLA-TiO_2_ composite and assessed its performance in adsorbing methyl orange and hexavalent chromium from aqueous solutions. The composite material was thoroughly characterized using techniques such as Fourier-transform infrared spectroscopy, scanning electron microscopy, Energy-dispersive X-ray, and X-ray diffraction. Batch adsorption experiments were conducted, and key parameters such as contact time, pH, adsorbent dosage, and concentration were varied systematically. The adsorption of MO and Cr(VI) fit the pseudo-second-order kinetic model and the Langmuir and the Freundlich isotherm models. The maximum adsorption capacity for MO was 277.7 ± 1.8 mg/g, and for Cr(VI), it was 33.98.3 ± 0.48 mg/g. The artificial neural networks model demonstrated a high coefficient of determination (R^2^ = 0.9996) and a low mean squared error (0.025), indicating its robustness in simulating the MO removal process under various conditions. Furthermore, the adsorption kinetics were well-described by Haldane’s model, which showed the best fit compared to other models tested. Notably, the GO@Cs-GLA-TiO_2_ composite was highly reusable, maintaining 85 ± 4.6% of its Cr(VI) adsorption capacity and 88.13 ± 3.05% of its MO adsorption capacity after four cycles of adsorption–desorption. This work highlights the significant potential of the GO@Cs-GLA-TiO_2_ composite as an efficient, sustainable material for wastewater treatment, making it a valuable contribution to environmental remediation research.

**Supplementary Information:**

The online version contains supplementary material available at 10.1186/s13065-025-01542-x.

## Introduction

In recent times, environmentalists have grown increasingly alarmed by the presence of harmful dyes in effluents, citing their adverse impacts on diverse life forms. The discharge of these dyes into the environment raises significant aesthetic and toxicological concerns [1]. Notably, industries such as chemical manufacturing, printing, dyeing, textile production, food processing, cosmetics manufacturing, and paper production are major contributors to colored effluents. Collectively, these industries release over 105 commercially available dyes, amounting to more than 7 × 10^5^ tons annually [2]. Methyl Orange (MO), a widely used anionic dye within the azo group, is classified as a type of *p*-amino azobenzene (p-AAB). It finds extensive application across various industries and research facilities, primarily as an acid–base indicator [3]. However, the discharge of MO and similar dyes presents significant challenges to the scientific community, as it constitutes one of the most hazardous environmental problems [4]. The persistence of these dyes in water bodies is particularly concerning, as they can remain for prolonged periods, disrupting vital processes such as photosynthesis [5], sunlight penetration, and dissolved oxygen levels. Additionally, they diminish the recreational value of water bodies and hinder the growth of aquatic biota [6]. The concentration of dyes in textile effluents varies significantly across different studies and industries. Laing, [7] indicated that dye levels typically range from 10 to 50 mg/L, serving as a common baseline for many textile industries. In Iraq, [8] found dye concentrations from 14 textile industries ranging from 20 to 50 mg/L, and [9] noted an outflow concentration of 45 mg/L for acid orange 10 from a textile factory in India [10]. Indicated that dye concentrations discharged from dye houses can vary from 10 to 250 mg/L. According to the Environmental Protection Agency, the permissible Cr(VI) concentrations in industrial wastewater and drinking water are 0.1 mg/L and 0.05 mg/L, respectively[11]. Çetin et al. [12] reported that the maximum chromium concentration in dyeing wastewater was approximately 2.7 mg/dm^3^. [13] found that Cr(VI) concentrations ranging from 1.05 to 1.86 mg/dm^3^ in seven textile mills’ raw textile wastewater in Pakistan. Chockalingam et al. [14] found that Cr(VI) levels in real effluents from the Sumukh textile mill in India varied between 1.16 and 2.2 mg/dm^3^. Al-Kdasi et al. [15] reported Cr(VI) concentrations between 0.5 and 0.6 mg/dm^3^ in dye wastewater from the Penfabric mill in Malaysia. Furthermore, [16] found that the average concentration of Cr (VI) in tannery effluents from the Hazaribagh area in Bangladesh was 374.19 mg/L, while in Nowapara, Jashore, it was 27.65 mg/L. Several studies [6, 11] emphasize the urgent need to address the challenges heavy metals and colored effluents to mitigate water scarcity and preserve environmental quality. To tackle the issue of non-biodegradable metals and harmful dyes in wastewater, various treatment methods have been employed, including oxidation, ozonation [17], photochemical processes [18], membrane filtration [19], ion exchange [19], coagulation/flocculation [20], electrochemical treatments [21], and biological degradation [22]. However, these conventional techniques are often prohibitively expensive on a large scale [23]. To address this, researchers have focused on developing more efficient composite materials by modifying their original forms to improve encapsulation of organic compounds or derivatives, thereby enhancing pollutant removal from surface and industrial wastewater [24]. Nonetheless, in many cases, functional modifications can reduce the specific surface area and the availability of strong active sites, thereby hindering regeneration and overall efficiency [5]. Hence, when designing adsorbents, it is imperative to consider the influence of surface area and active site availability on adsorption performance [6]. Recycling adsorbents remains a major challenge in adsorption technology [25]. Adsorption in the solution phase has emerged as a widely adopted method for removing organic dyes due to its simplicity, cost-effectiveness, and high efficiency. However, traditional sorbents such as activated carbon [26], zeolites [27], biomaterials [28], and polymers often fall short in terms of efficiency [29]. To address this limitation, extensive research efforts have been directed towards finding more effective adsorbents. Various adsorbents have been developed and utilized for the rapid removal of harmful impurities from aqueous solutions, including carbon nanotubes [30, 31], multi-walled carbon nanotubes [32], nanoparticles [33], nanocomposites [34], rubber tire [35], and other low-cost materials [4, 36–41]. Nanostructured materials, especially those based on MgO, TiO₂, and Fe₃O₄, as well as their nanocomposites, have gained considerable attention due to their abundance and environmentally friendly nature [11]. These materials exhibit high catalytic performance, attributed to their large surface areas and unique pore structures. Despite efforts to enhance their adsorption capacity through controlled crystallite growth and the creation of highly porous structures, practical applications remain limited [42]. Titanium dioxide (TiO₂) is one of the most studied metal oxide semiconductors due to its excellent photocatalytic activity under UV light [43]. During dye wastewater treatment, TiO₂ particles tend to aggregate, forming fine clusters that hinder material reuse and recovery, thereby increasing operational costs. As such, our strategy focuses on developing nanocomposite materials based on graphene oxide and TiO₂ to enhance the adsorption capacity for MO dyes [44]. Graphene Oxide (GO) consists of two-dimensional graphene sheets with sp^2^ hybridized carbon atoms and contains oxygenated functional groups and structural defects [42]. It can exist as single-, double-, or few-layer sheets, with a single layer being only 0.335 nm thick [45]. This unique nanostructure endows GO with exceptional physicochemical properties, including high electron mobility [46], optical transparency [47], thermal conductivity, large specific surface area [6], and chemical stability [48]. Its hydrophilicity arises from the presence of carboxyl, carbonyl, and hydroxyl groups [49]. Three-dimensional graphene structures are created by combining two-dimensional GO sheets with TiO₂ nanoparticles [50]. This integration increases the surface area available for adsorption and provides additional pathways for charge transport, thereby reducing electron–hole recombination [51]. Achieving a uniform distribution of TiO₂ on GO sheets significantly improves adsorption efficiency. However, inorganic nanomaterials are inherently small and pose recycling challenges in water treatment systems [52]. Chitosan plays a crucial role in preventing nanomaterial aggregation and promotes catalytic reactions at their surfaces [52]. Natural polysaccharide composites, known for their rich pore structures, excellent hydrophilicity [53], non-toxicity [54], and strong adsorption capabilities [20], offer great potential for wastewater treatment. Chitosan’s hydroxyl (-OH) and amino (-NH₂) groups enhance its capacity to adsorb both anionic dyes and metal ions effectively [20]. This research focuses on the development of a novel, chemically stable adsorbent composed of cross-linked chitosan-GO-TiO₂. The primary goal is to increase the number of active sites within the polymer structure, resulting in a multifunctional composite capable of efficiently adsorbing methyl orange dye from wastewater while also demonstrating antibacterial activity. The Cs/GA composite hydrogel offers several advantages, including low cost, enhanced mechanical strength, and improved stability. Functional groups like -COO⁻ and -OH on rGO-TiO₂ enable its uniform dispersion and stabilization within the porous structure of the Cs/GA matrix [1]. Immobilizing microorganisms on nano-sized materials enhances degradation efficiency and enables easy recovery and reuse, thereby reducing costs and environmental impacts. However, data on the biodegradation kinetics and the prediction of residual concentrations of MO degradation by the biocomposite fungal Cs-GLA-GO and associated microbial growth remain limited. This research proposes the development of a low-cost, eco-friendly, reusable, and stable nanocomposite adsorbent composed of cross-linked chitosan-GO-TiO₂. The primary aim is to increase the number of active sites in the polymer matrix, thus creating a multifunctional material capable of simultaneously adsorbing methyl orange dye and Cr(VI) from aqueous solutions. A secondary objective is the immobilization of *Trichoderma* sp. onto the Cs-GLA-GO matrix to facilitate both removal and degradation of MO. To achieve these goals, the composite materials were synthesized and thoroughly characterized using various analytical techniques, including Fourier-transform infrared spectroscopy (FTIR), scanning electron microscopy (SEM), and X-ray diffraction (XRD). Subsequently, the effects of various parameters on MO adsorption were studied. This included analyzing adsorption kinetics, isotherm models, thermodynamics, regeneration processes, and computational studies to better understand the adsorption mechanism. Furthermore, an artificial neural network (ANN) and response surface methodology (RSM) models were developed to predict process efficiency.

## Materials and methods

### Materials

Titanium(IV) oxide (reagent grade, Merck, Germany), glutaraldehyde (GLA), and methyl orange dye were purchased from Sigma-Aldrich (USA). Chitosan (Cs) (deacetylation ≥ 90%; medium molecular weight) was obtained from Piochem (Egypt), while graphite was procured from Loba Chemie (India). Acetic acid, absolute ethanol, sodium hydroxide (NaOH), and hydrochloric acid (HCl) were purchased from Merck (Egypt).

### Fabrication of Cs-GLA-TiO_2_- GO

Desorption and regeneration experiments were conducted after synthesizing GO@Cs–GLA–TiO₂ using a modified Hummers’ method [4].

- 1. *Graphene Oxide (GO)* GO was synthesized using a modified Hummers method. Graphite powder (1.7 g) was mixed with concentrated H₂SO₄ (45 mL) and H₃PO₄ (100 mL) in an ice bath. Potassium permanganate (KMnO₄, 5 g) was added gradually under constant stirring while maintaining the temperature at 10 °C for 4 h. The mixture was then stirred overnight at 50 °C, resulting in a color change from black to brown. Water (60 mL) was carefully added to the reaction mixture, followed by the addition of 30% H₂O₂ (50 mL) to quench the reaction. The resulting yellow suspension was centrifuged and washed with 15% HCl, ethanol(C₂H₅OH), and deionized water until a neutral pH was achieved. The final product was dried at 40 °C for 72 h.
- 2. *Chitosan (Cs)* Chitosan powder was dissolved in a 20% v/v aqueous (CH₃COOH) acetic acid solution by stirring overnight to form a 2 wt% chitosan solution.
- 3. *Titanium Dioxide (TiO₂)* TiO₂ nanoparticles (2 wt%) were dispersed in the chitosan solution under constant stirring for 2 h to obtain the Cs-TiO₂ nanocomposite.
- 4. *Glutaraldehyde (GLA)* GLA was used for cross-linking. The GO solution was mixed with an aqueous Fe^3^⁺/Fe^2^⁺ solution (3.9 g FeCl₃·6H₂O and 2.7 g FeCl₂·4H₂O in 100 mL of water). The mixture was stirred for 5 h and cross-linked with 2% glutaraldehyde (GLA) for 2 h at 40 °C. The final composite was washed, dried at 60 °C overnight, and further characterized using SEM, XRD, and FTIR analysis, as shown in Table S1.

### Strain isolation and preparation of biomass

An isolate of *Trichoderma sp* strain, sourced from El-Gahrbia drain, served as the inoculum at various concentrations for the degradation of a mixture of MO throughout the research. The 18S rDNA sequences of this fungal strain were deposited in GenBank under accession number PQ462654.1, as shown in Fig. S1. The sequence data for *Trichoderma viride* isolate MT, including the small subunit ribosomal RNA gene (partial), internal transcribed spacer 1, 5.8S ribosomal RNA gene, internal transcribed spacer 2 (complete), and large subunit ribosomal RNA gene (partial), have been deposited in GenBank under the accession number PQ462654.1 and are publicly available at: https://www.ncbi.nlm.nih.gov/nuccore/PQ462654.1.

Microbial growth and biodegradation experiments were conducted using 150-ml conical flasks. The flasks were prepared with a solution consisting of 0.2 g per liter of dipotassium orthophosphate (K_2_HPO_4_), 0.8 g per liter of potassium dihydrogen orthophosphate (KH_2_PO_4_), 0.2 g per liter of magnesium sulfate (MgSO_4_), 0.1 g per liter of calcium chloride (CaCl_2._6H_2_O), 0.1 g per liter of ammonium sulfate (NH_4_)_2_SO_4_, 0.2 g per liter of yeast extract, and 3 g/Lof glucose.

The preparation of hydrogel began with the dissolution of 1 g of sodium alginate in 100 mL of distilled water, heated for a period, and then sterilized via autoclaving. Following sterilization, the sodium alginate was mixed with 4 g of fungal pellets, then mixed with Cs-GLA-TiO_2_-GO, then the mixture was transferred into a pour in calcium chloride.

### Adsorption experiments

In our adsorption studies, MO and Cr(VI) solutions were prepared by dissolving precise amounts of MO and (K_2_​Cr_2_​O_7_​) potassium dichromate in ultrapure water. These stock solutions were then appropriately diluted to achieve the desired concentrations before utilization. Following optimized parameters, batch sorption experiments were conducted in duplicate. Each experiment involved adding 0.4 g/L of the prepared adsorbent into a 100 mL conical flask containing 100 mL of targeted contaminants with initial concentrations of 20/10 mgL^−1^ at a specific solution pH. The mixture was thoroughly mixed on a mechanical shaker at 180 rpm. Various parameters such as dosage effect (ranging from 0.2 g to 1 g per 100 mL), contact time (varying from 10 to 100 min), solution pH (ranging from 2 to 7), and temperature effect (ranging from 298 to 318 K) were manipulated to ascertain the optimal conditions for MO dye adsorption and Cr(VI). Post adsorption, the mixture was filtered using 0.22 mm filters, and residual concentrations of MO and Cr(VI) were determined using a UV–vis spectrophotometer at 464 nm and ICP-MS (Perkin Elmer). Additionally, we investigated the influence of reaction time (ranging from 0 to 100 min), solution pH (ranging from 2 to 7), and regeneration on the adsorption of MO and Cr(VI). Adsorption isotherm experiments were conducted at initial concentrations of MO/Cr(VI). (initial concentration: 20/10 mgL^−1^; temperature = 25 °C; adsorbent dosage 0.4 g/L). We used Eqs. (1) and (2) to calculate the adsorption capacity and percentage.

1 $$\text{Biodecolourization \%}=\frac{B^\circ -B}{B^\circ }\times 100$$

2 $$qe=\frac{(Ci-C)}{M} V$$

where B° and B are the absorbance of the concentrations of the dye before and after decolourization respectively. Ci and Co refer to the initial concentration of pollutants and the equilibrium concentration after the application of the adsorbent, measured in mg/L. Additionally, V and M represent the volume of the solution in mL and the mass of the adsorbent in grams, respectively.

### Experimental design and statistical analysis

A statistical approach called response surface methodology (RSM) was employed to study the elimination of dye. The Box-Behnken design (version 13 of Design-Expert) allows for the simultaneous analysis of multiple factors and their interactions while using a limited number of observations. This approach aids in optimizing process parameters to achieve the highest response, such as yield or predicted adsorption capacity. The study examines the influence of four independent variables: initial dye concentration, adsorbent dosage, contact time, and solution pH, as detailed in Table S2. A quadratic second-degree polynomial equation is employed for approximation, represented in Eq. (3). [38]. This study used a quadratic second-degree polynomial equation for approximation and can be expressed in Eq. (3).

3 $${\text{R}} = { }K_{^\circ } { } + \mathop \sum \limits_{i = 1}^{n} K_{{\text{i}}} {\text{ V}}_{{\text{i}}} { } + \mathop \sum \limits_{{{\text{i}} = 1}}^{{{\text{n}} - 1}} \mathop \sum \limits_{j = 1 + 1}^{n} {\text{K}}_{{{\text{ij}}}} {\text{ V V}}_{{\text{j}}} + { }\mathop \sum \limits_{i = 1}^{n} K_{{{\text{ij}}}} {\text{ V}}_{{\text{i}}}^{2} + \varepsilon$$

### Development of an artificial neural network predictive model for flocculation reactions

**Fig. 1:**
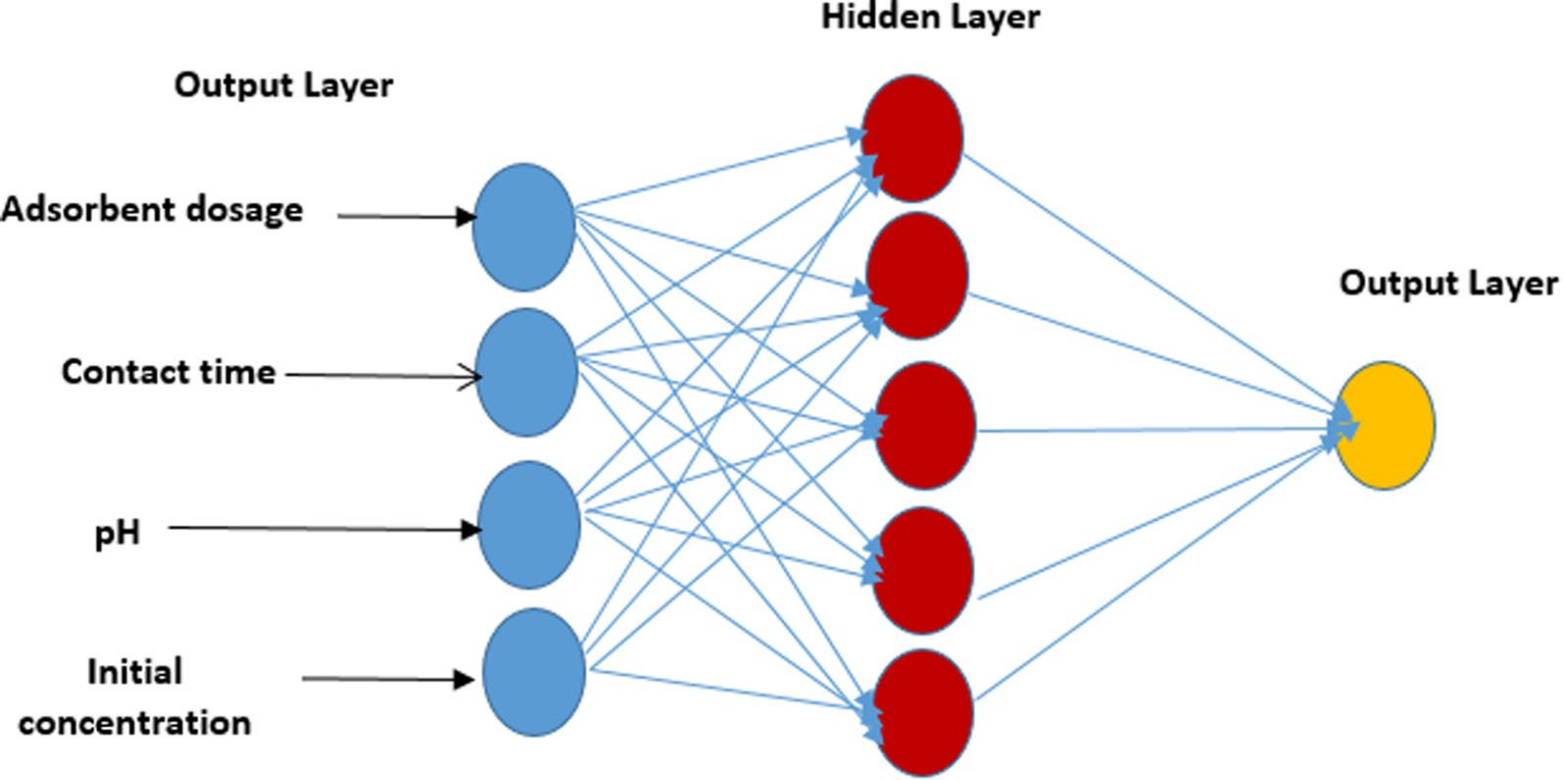
ANN topology for the adsorption of MO onto the GO @Cs-GLA-TiO_2_ composite

In this study, MATLAB was utilized for predicting adsorption efficiency using an Artificial Neural Network (ANN). ANNs are renowned for their ability to effectively capture nonlinear effects, making them highly applicable in scenarios where a relationship, albeit highly nonlinear, exists between independent and dependent variables [55]. The ANN architecture consisted of three layers: an input layer with 4 neurons representing initial pH, adsorbent dosage, contact time, and intimal concentration MO; and a total of 29 sets of data for predicting the removal rate of MO. The artificial neural network (ANN) model consists of four neurons in the input layer, five neurons in the hidden layer, and one neuron in the output layer. The basic structural diagram of this ANN is illustrated in Fig. 1. In this study, a multilayer perceptron feed-forward neural network was utilized alongside a back-propagation algorithm, Levenberg Marquardt Algorithm and a log-sigmoid activation function to model and predict the efficiencies of GO@Cs-GLA-TiO_2_ in adsorbing MO [55]. Data derived from response surface methodology (RSM) analysis were split into 15% for training, 15% for testing, and 70% for validation. During training, the network adjusted neuron weights to achieve the best fit in terms of coefficient of determination (R^2^) and (RMSE) values. Predictions were subsequently made using the optimized model [56].

## Results and discussion

### Scanning electron microscopy (SEM)

**Fig. 2:**
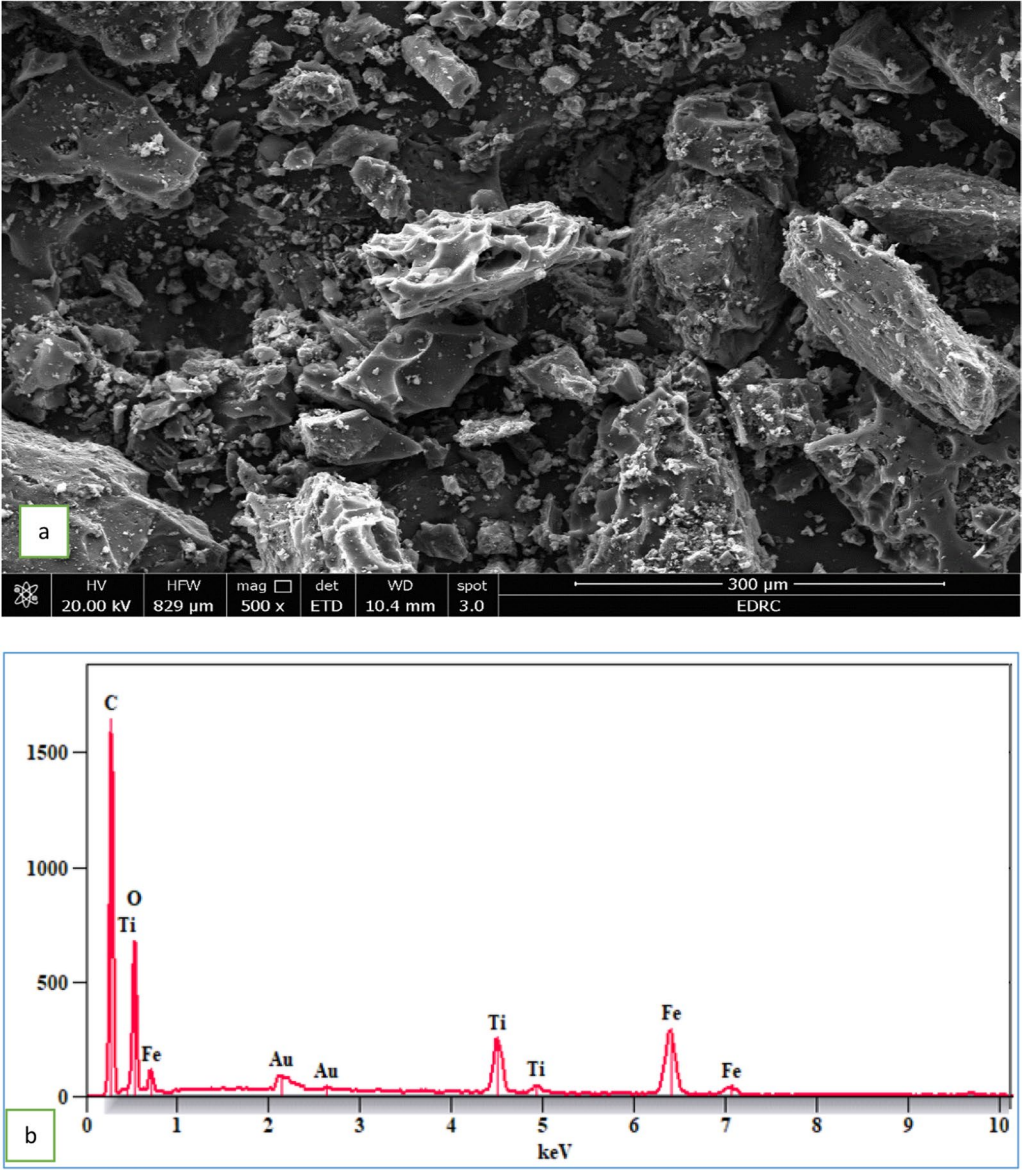
**a** SEM, **b** EDX analysis of nano composite

Figure 2a presents SEM images recording the texture and morphology of GO@Cs-TiO_2_ composites. The image demonstrates that graphene oxide sheets are dispersed among iron(III) oxide hydroxide nanoparticles. A homogeneous dispersion of nanoparticles results from graphene oxide’s significant surface area. Fe(III) nanoparticles may interact with the functional groups on the graphene oxide surface, such as COOH, CO, and OH, leading to the wrinkled surface. But after chitosan immobilization, the surface becomes noticeably more rocky, rough, and wrinkled. Figure 2b presents the EDX analysis of graphene oxide/chitosan/iron(III) oxide hydroxide, indicating 25.3 wt% iron, 29.1 wt% carbon, 26.8 wt% oxygen, 10.8 wt%TiO_2_, and 1.1 wt% nitrogen. The FT-IR spectra were recorded in the range of 500–4000 cm⁻^1^, as illustrated in Fig. S2a. In Fig. S2b, the FT-IR spectra of graphene oxide are presented. The absorption peak at 3334.45 cm⁻^1^ is attributed to O–H stretching, while the peak at 2224.46 cm⁻^1^ corresponds to C≡C stretching vibrations. Additionally, the C = O stretching vibrations of the carbonyl group can be observed around 1970.45 cm⁻^1^ [57]. The characteristic peak at 1609.00 cm⁻^1^ is associated with C = C stretching vibrations. Peaks at 1012 cm⁻^1^ and 1427.67 cm⁻^1^ are linked to carbon–oxygen bonds from hydroxyl and carboxyl groups. In the composite material, peaks in the range of 3879.31, 3887.99 cm^1^ correspond to O–H stretching vibrations from hydroxyl groups, likely derived from chitosan and iron(III) oxide hydroxide (FeOOH). The peak at 3425.94 cm⁻^1^ is related to N–H stretching vibrations of amino groups in chitosan. The peak at 2345.14 cm⁻^1^ may indicate C = O stretching vibrations from carbonyl groups, possibly due to the oxidation of graphene. Peaks in the range of 2127.62 cm⁻^1^, 2069.19 cm⁻^1^, 2019.97 cm⁻^1^, 1971.51 cm⁻^1^ are attributed to C≡C stretching vibrations in the graphene oxide structure. The peak at 1889.39 cm⁻^1^ corresponds to C = O stretching vibrations in carbonyl groups. The peak at 1636.30 cm⁻^1^ is associated with N–H bending vibrations of amino groups in chitosan, while the peak around 1544.05 cm⁻^1^ likely results from C = C stretching vibrations in the aromatic rings of graphene oxide. The peak at 1403.49 cm⁻^1^ could be due to C-O–H bending vibrations of hydroxyl groups in the composite [58]. The peak at 1121.95 cm⁻^1^ corresponds to C–O–C stretching vibrations of epoxy groups in graphene oxide, and the peak at 1018.15 cm⁻^1^ is associated with C-O stretching vibrations of alkoxy groups in the composite. The peak at 762.06 cm⁻^1^ may indicate Fe–O vibrations from the iron(III) oxide hydroxide component, while the peak at 623.71 cm⁻^1^ could be attributed to Ti–O vibrations from the TiO_2_ component. Finally, the low-frequency peak at 444.24 cm⁻^1^ might be related to metal–oxygen vibrations in the composite, such as Fe–O or Ti–O [59]. From the perspective of adsorbed Cr(VI), the symmetric tensile vibration of the N–H bond i NH2 at 3153.02 cm⁻^1^ showed a significant reduction. Similar changes were observed in bands at 1630 cm⁻^1^, 1544.05 cm⁻^1^, and 1403.49 cm⁻^1^, which correspond to the N–H bending vibrations, C = C stretching vibrations, and C-O–H bending vibrations, respectively. These alterations confirm the adsorption of Cr(VI) onto the composite, with the Fe\O peak shifting to 547.18 cm⁻^1^. This suggests that both the –NH2 group and Fe\O are involved in the removal of Cr(VI) [5]. In the case of methyl orange (MO), it is noteworthy that the intensity of the peaks in the region of 3334.45 cm⁻^1^ (associated with –OH stretching vibrations) increased [60]. Additionally, after loading MO onto the nanocomposite, both the increase and shift of peaks were noted. The prominent peaks detected post-adsorption were identified at 2348.71 cm⁻^1^, 2224.47 cm⁻^1^ (C≡C), 1970.45 cm⁻^1^ (C = O), 1609.00 cm⁻^1^ (C = C), 1427.67 cm⁻^1^ (C–O), and 1012.20 cm⁻^1^ (C–O). Overall, the changes in peak formation, reduction, and shifting observed after adsorption indicate a strong interaction between methyl orange and GO@Cs-TiO_2_ [3].

### The X-ray diffraction (XRD)

**Fig. 3:**
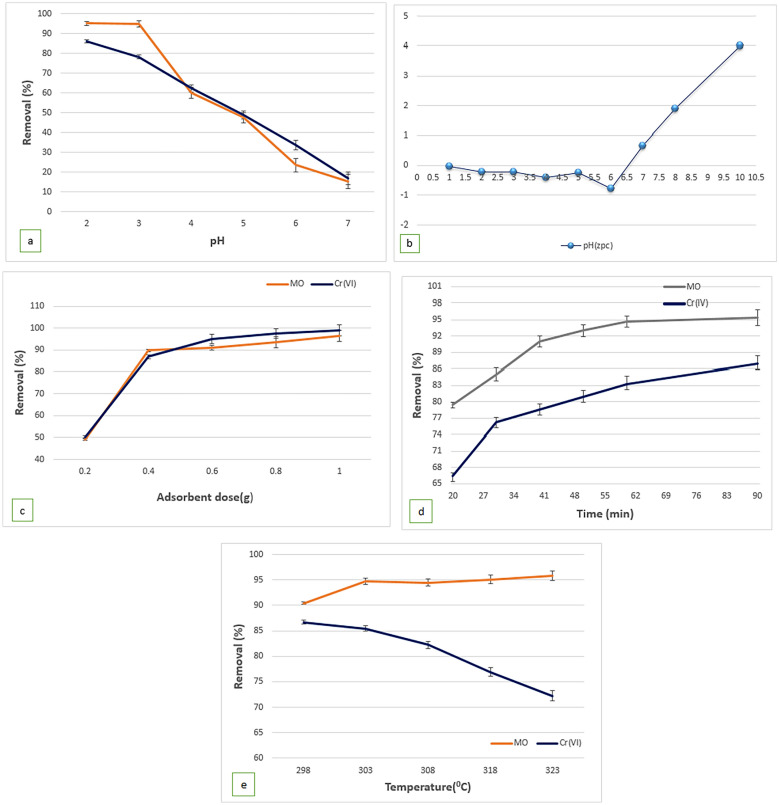
Effect of **a**, **b** pH, **c** absorbed dose, **d** reaction time, **e** initial concentration and, **f** temperature on the MO/Cr(VI)

The X-ray diffraction (XRD) pattern of GO@Cs-GLA-TiO_2_ is shown in Fig. S3a. A peak at 2θ = 29.93° corresponds to the graphene oxide structure, indicating an interlayer distance of 3.6 Å. Additionally, a peak at 2θ = 32.02° with an interlayer distance of 5.88 Å suggests the presence of a small quantity of non-oxidized graphite. This shift in interlayer distance, from 3.6 Å to 5.88 Å, indicates the incorporation of oxygen-containing functional groups and an increase in the thickness of the graphite sheets. Fig. S3b presents the XRD pattern of graphene oxide/chitosan/iron(III) oxide hydroxide nanocomposites. By comparing the pattern to the standard JCPDS card (074–3080), peaks at 2θ = 19.20°, 20.19°, 26.20°, 30.16°, 35.64°, 38.12°, 43.03°, 52.23°, and 63.22° are attributed to the goethite crystal phase. Figure 3Sc shows the XRD spectrum of TiO_2_, where the diffraction peaks correspond to the anatase phase (JCPDS file 71–1166), with distinct peaks at 2θ = 25.13°, 37.46°, 38.04°, 48.71°, 54.63°, 55.33°, 63.54°, and 70.02°. Additionally, the peaks at 2θ = 12.08° and 22.02° are attributed to chitosan, as depicted in Fig. S3d. The analysis of the interlayer distance confirms that the inclusion of chitosan did not significantly affect the interlayer spacing. Therefore, it can be concluded that chitosan is exfoliated and encapsulates the graphite sheets.

### Influence of operational parameter pH on MO/Cr(VI) adsorption

The operational parameters, namely pH, contact time, dosage, and concentration, significantly affect the adsorption of MO/Cr(VI). Solution pH plays a crucial role in controlling the surface properties of GO@Cs-GLA-TiO_2_ and the ionization of the adsorbate in the aqueous medium [61]. According to [62], the chromium concentrations in untreated textile wastewater are typically found within a pH range of 3.85 to 7.8. To investigate the influence of pH on the removal efficiency of MO and Cr(VI), experiments were conducted over a pH range of 2.0 to 7.0. Using 0.4 g/L of GO@Cs-GLA-TiO_2_, an adsorbate concentration of 20/10 mg/L, and a contact time of 90 min at 25 °C, the removal percentages (% R) were determined, as depicted in Fig. 3a. The predominant anionic species of hexavalent chromium in solution, occurring within the pH range of 1.0 to 6.0, is HCrO^4−^ [63]. The highest removal percentages for both Cr(VI) and MO were observed at pH 2.05 (89.08 ± 0.9% for Cr(VI) and 95.3 ± 0.6% for MO at pH 3), with values decreasing as pH increased. At lower pH values, the GO@Cs-GLA-TiO_2_ surface carried a positive charge due to protonation of functional groups (e.g., OH^+^, NH^3+^, and Fe–OH^2+^), facilitating interactions with the negatively charged anionic forms of MO and HCrO^4−^ [60], thus maximizing removal percentages. As pH increased, the abundance of hydroxyl ions in the solution and the negativity of active adsorption sites augmented, leading to repulsion of Cr(VI) and MO species and consequently decreasing the removal efficiency of GO@Cs-GLA-TiO_2_. The adsorption percentage is higher when using a composite of graphene oxide/chitosan/iron(III) oxide hydroxide. This is explained by the existence of chitosan and oxygen-functional groups in graphene oxide [57]. Apart from hydrogen bonding, various interactions such as n–π and π–π were anticipated to be involved in MO adsorption, resulting in an enhanced removal percentage of MO compared to Cr(VI) [64]. Consequently, pH 2.05 and pH 3 were chosen for all MO and Cr(VI) adsorption experiments. Zeta potential measurements of GO@Cs-GLA-TiO_2_ revealed variations in response to pH changes in the dispersion solution, as shown in Fig. 3b. Notably, assessment of the surface charge of GO@Cs-GLA-TiO_2_ through zeta potential measurement at different pH values indicates a point of zero charges (pHpzc) of 6.01 (Fig. 3b). Below the pHpzc, GO@Cs-GLA-TiO_2_ exhibits a positive surface charge at solution pH 2.05 and 3, leading to electrostatic attraction between MO and Cr(VI) and GO@Cs-GLA-TiO_2_[65].

### Effect of contact time

The impact of reaction duration on the efficiency of MO and Cr(VI) ion adsorption by the GO@Cs-GLA-TiO_2_ composite was explored over varying time intervals. Illustrated in Fig. 3c, the GO@Cs-GLA-TiO_2_ hybrid displayed the highest adsorption rate among the mentioned adsorbent materials for both pollutants. The removal capacity surged rapidly, gradually tapering off until saturation was reached around the 90-min mark. Initially, within the first 10 min, the removal efficiency was measured at 79.04 ± 0.2% for MO and 66.4 ± 0.3% for Cr(VI) ion, indicating swift adsorption onto the GO@Cs-GLA-TiO_2_ composite. This rapid adsorption can be attributed to the strong interaction between the adsorbates and numerous reactive binding sites on the adsorbent surface. The adsorption of MO/Cr(VI) onto GO@Cs-GLA-TiO_2_ exhibited an increasing trend with longer contact times, stabilizing at sorption equilibrium after 40 min. The removal efficiency achieved was 93.03 ± 0.51% and 80.01 ± 0.54%, respectively. As the adsorption progressed, the rate slowed down, reflecting the occupation of accessible reactive sites on the adsorbent surface. Nonetheless, the GO@Cs-GLA-TiO_2_ composite, with its array of reactive functional groups, including carboxyl and Ca^2+^, could effectively interact with the negatively charged pollutants via strong electrostatic forces of attraction. These results prompted further investigation solely on the GO@Cs-GLA-TiO_2_ hybrid composite, with an observed equilibrium time of approximately 90 min for the adsorption of MO and Cr(VI) onto this composite[64, 65].

### Effect of adsorbent dosage on MO and Cr(VI) adsorption

The investigation conducted to assess the impact of varying adsorbent quantities (0.2, 0.4, 0.6, 0.8 g, and 1 g/L) on the adsorption of MO and Cr(VI) is illustrated in Fig. 3d. With an initial MO/Cr(VI) concentration of 20/10 mg/L, respectively, pH values set at 3 and 2.05, a temperature of 25 °C, and a contact time of 90 min, the study revealed a noticeable increase in the adsorption of both MO and Cr(VI) with the escalation in the amount of adsorbent. The removal efficiency of both MO and Cr(VI) showed significant improvements, with the former rising from roughly 85.1 ± % 0.3 to 98.9 ± 0.15%, while the removal efficiency for Cr(VI) increased from and the latter from 61.1 ± 0.55% to 96.3 ± 0.51%. This augmentation can be attributed to the expanded contact surface area between the adsorbent and pollutants, resulting in the availability of more adsorption sites on the surface. Consequently, the adsorption potential of GO@Cs-GLA-TiO_2_ composites for dye pollutants is enhanced, leading to an overall improvement in dye removal efficiency, a trend supported by prior studies [66]. However, beyond a certain threshold, the adsorption capacity diminishes, likely due to the saturation of adsorption sites, leading to overcrowding of adsorbent particles [2]. Furthermore, increasing the initial MO/Cr(VI) concentration from 20 to 200 mg/L and 10 to 100 mg/L, respectively, at 25 °C, 90 min, with 0.4 g of adsorbent and optimum pH of 3 and 2.05, resulted in an elevation of sorption capacity for MO from 19.7 ± 0.5 to 199.05 ± 1.04 mg/g and Cr(VI) from 2.44 ± 0.07 to 24.83 ± 0.4 mg/g for GO@Cs-GLA-TiO_2_, respectively. At lower concentrations of MO/Cr(VI), the ratio of available binding sites on the adsorbents is higher compared to the initial amount of MO/Cr(VI). However, at elevated concentrations, there are fewer adsorption sites available. The significant adsorption capacity noted with 0.4 g/L of both adsorbents is due to the easy access to free active sites on their surfaces. Nonetheless, as the adsorbent dosage increases, the number of active sites may rise, but the ratio of MO/Cr(VI) to active sites decreases, leading to a gradual decline in adsorption capacity[67].

### Effect of temperature on MO and Cr(VI) adsorption

The potential of GO@Cs-GLA-TiO_2_ nanocomposites in the adsorption of MO/Cr(VI) pollutants, with initial concentrations of 20/10 mg/L, under varying solution temperatures (25–50 °C), an adsorbent amount of 0.4 g/L, pH values set at 3 and 2.05, and a contact time of 90 min, was investigated, as depicted in Fig. 3e. Notably, temperature changes exert a significant influence on the removal efficiency of MO dye. A slight increase, from approximately 90.4 ± 0.9% to 94.7 ± 0.49%, in the removal efficiency of the MO dye was observed with a rise in temperature from 25 to 50 °C. This increase can be attributed to the enhanced mobility of dye molecules and their improved access to the small pores of the nanocomposite at higher temperatures, consequently augmenting the adsorption of the dye [53]. Specifically, for MO dye, adsorption increases as temperature rises from 25 to 30 °C. However, beyond 50 °C, a further increase in temperature weakens the interaction between adsorption sites on the absorbent surface and dye molecules, leading to a decline in MO adsorption [5]. Therefore, the optimal temperature for MO adsorption by the GO@Cs-GLA-TiO_2_ composite was considered to be 30 °C. Conversely, a diminishing trend in the percentage of Cr(VI) removal was observed as the solution temperature increased from 298 to 318 K, decreasing from 86.5 ± 0.2% to 72.24 ± 0.4%, respectively. These outcomes suggest a linear decrease in Cr(VI) adsorption on the nanocomposites with increasing adsorption temperature. The study indicates that Cr(VI) removal is favored at lower temperatures due to the involvement of stronger interaction forces during the adsorption process. However, as temperature rises, these adsorptive forces weaken, leading to a breakdown and consequent decrease in Cr(VI) removal at higher temperatures [60].

### Adsorption kinetics

**Fig. 4:**
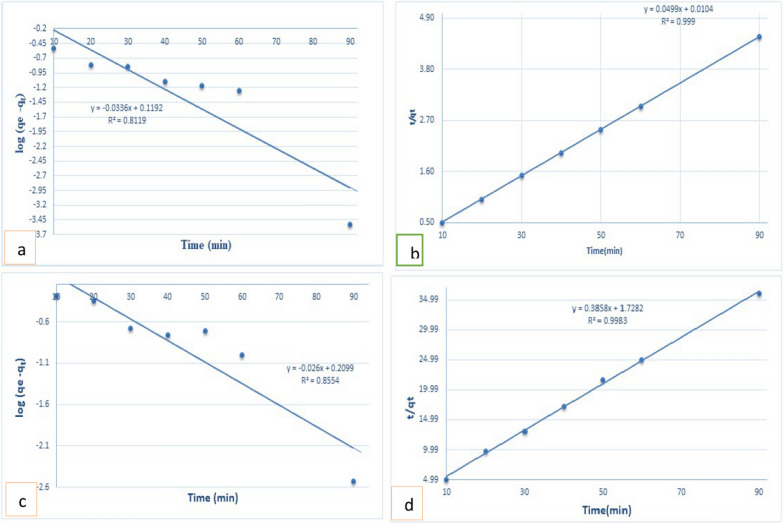
Adsorption kinetics of for (**a**, **c**) MO and (**b**, **d**) Cr(VI) adsorption on GO@Cs-GLA-TiO_2_

The investigation involved the use of various models, including pseudo-1st-order, pseudo-2th-order, intra-particle diffusion, and Elovich Eqs. (4 to 7) in Table S3 [50], which were utilized to analyze experimental data and discern the mechanism and rate-controlling step of adsorption. Figure 4 illustrates the results of kinetic parameters, and Table 1 provides a detailed list. The q_e_ and q_t_ are the quantities of the adsorbed MO/Cr(VI) (mg/g) at equilibrium and time t, respectively, and k_2_ is the adsorption rate constant (g/mg.min). α is the initial adsorption rate (mg g^−1^ min^−1^), β is the adsorption constant, b is the rate constant, and kf is the rate coefficient value (mg g^−1^ min^−1^). Notably, the experimental data exhibited superior fitting with the pseudo-2nd-order model (PSO) (R^2^ = 0.999/0.998) compared to the pseudo-1st-order model (R^2^ = 0.81/0.85) for the developed adsorbent depicted in Fig. 4a, b. Evaluation based on the pseudo-first-order model yielded a calculated qe value of MO/Cr(VI) 1.31 ± 0.05 mgg^−1^/1.621 ± 0.01 mgg^−1^, respectively, which significantly deviated from the experimental value of 19.99 ± 0.06 mgg^−1^/2.49 ± 0.08 mgg^−1^, respectively. Furthermore, the experimental adsorption capacity values closely matched the calculated ones, with a calculated qe value of MO/Cr(VI) 20.04 ± 0.062 mgg^−1^/2.54 ± 0.05 mgg^−1^, respectively, much akin to the experimental data of 19.99 ± 0.06 mgg^−1^/2.49 ± 0.08 mgg^−1^, respectively for GO@Cs-GLA-TiO_2_, as shown in Fig. 4c, d and Table 1. The dominance of the PSO model in the adsorption process implies chemisorption involving electron exchange and covalent forces, along with ion exchange [51]. The Elovich equation exhibited commendable fitting to the experimental data, with high (R^2^) values; this suggests that the adsorption process of MO/Cr(VI) by the GO@Cs-GLA-TiO_2_ exhibits energetically heterogeneous behavior as summarized in Table 1. In Table 1, the higher value of AE indicates a more available surface for MO/Cr(VI) on GO@Cs-GLA-TiO_2_, and a linear correlation coefficient (0.965 and 0.982) for MO/Cr(VI), respectively. On the other hand, the higher value of BE for GO@Cs-GLA-TiO_2_ shows a greater affinity in adsorption, which increased while desorption decreased. This observation suggests the heterogeneity of adsorbent surfaces and confirms the chemisorption process as predicted by the pseudo-second-order model. Based on the correlation coefficients (R^2^) in Table 1, the pseudo-second-order and Elovich models of GO@Cs-GLA-TiO_2_ composite adsorbent have higher R^2^ (0.999) than those obtained from the pseudo-first-order model.

**Table 1:** Kinetics parameters for the MO/Cr(VI) adsorption with GO@Cs-GLA-TiO_2_

Model	Kinetic parameter	GO@Cs-GLA-TiO_2_	
		MO	Cr(VI)
Pseudo-first-order	qe _cal_. (mg g^−1^)	1.31	1.62
	qe _exp_. (mg g^−1^)	19.9	2.49
	K_1_ (min^−1^)	0.077	0.05
	R^2^	0.81	0.85
Pseudo second-order	qe _exp_. (mg g^−1^)	19.9	2.49
	qe _cal_. (mg g^−1^)	20.04	2.54
	K_2_ (min^−1^)	0.08	0.09
	R^2^	0.999	0.998
Intraparticle diffusion	Stage 1 Kpi(mg/g.min^1/2^)	0.0967	0.183
	Ci	19.204	6.22
	R^2^	0.992	0.991
	Stage 2 Kpi(mg/g.min^1/2^)	0.0841	0.0742
	Ci	19.303	1.695
	R^2^	0.97	0.95
	Stage 3 Kpi(mg/g.min^1/2^)	0.077	0.0036
	Ci	19.385	3.62
	R^2^	0.80	0.79
Elovich equation	BE	10.92	1.744
	AE	0.27980	0.0792
	R^2^	0.991	0.982

### A mechanistic model for MO and Cr(IV) diffusion

The plot representing intra-particle diffusion revealed three distinct linear stages, as in Table 1. Stage 1 was attributed to film diffusion, occurring during the rapid transport of the adsorbate, MO/Cr(VI), from the bulk solution to the external surface of GO@Cs-Fe-GLA-TiO_2_. The gradual rise in adsorption (stage 2) and the reaching of equilibrium (stage 3) were probably due to pore diffusion mechanisms. A steeper slope suggests a quicker adsorption process. Analysis of the kpi parameter at each stage indicated that film diffusion (stage 1) was faster than pore diffusion (stages 2 and 3). The first stage obtained the most excellent linearization (R^2^ = 0.992) with corresponding stage (1) intraparticle rate constants (0.0967 ± 0.002 mgg^−1^ min^0.5^), indicating the most rapid process. The thickness value typically correlates with the degree of mass transfer resistance; thicker boundaries entail higher resistance. Therefore, the initial diffusion stage (stage 1) experienced the highest mass transfer resistance, likely due to the chitosan coating on the external surface [68]. This observation suggests that the MO/Cr(VI) solute initially interacts with the active sites of chitosan (NH_2_ and OH) on the external surface of GO@Cs-GLA-TiO_2_. As the external surface becomes almost saturated, the solute traverses the tortuous chitosan polymeric network to reach the internal pores of GO@Cs-GLA-TiO_2_. The diffusion rates of MO at stage (2) (0.0841 ± 0.002 mgg^−1^ min^0.5^) and stage (3) (0.056 ± 0.003 mgg^−1^ min^0.5^) were lower than that at stage (1) (0.0967 ± 0.002 mgg^−1^ min^0.5^), with corresponding lower R^2^ values (0.97 and 0.63), on the other hand, the diffusion rates of Cr(VI) at stage (2) (0.0747 ± 0.004 mgg^−1^ min^0.5^) and stage(3) (0.0036 ± 0.0001 mgg^−1^ min^0.5^) were lower than that at stage(1) (0.1883 ± 0.003 mgg^−1^ min^0.5^), with corresponding lower R^2^ values (0.95 and 0.79) Table 1, indicating that the diffusion of MO/Cr(VI) to mesoporous GO@Cs-GLA-TiO_2_ was expedited, likely due to the tendency to reach equilibrium, resulting in rapid uptake.

### Adsorption isotherm models

**Fig. 5:**
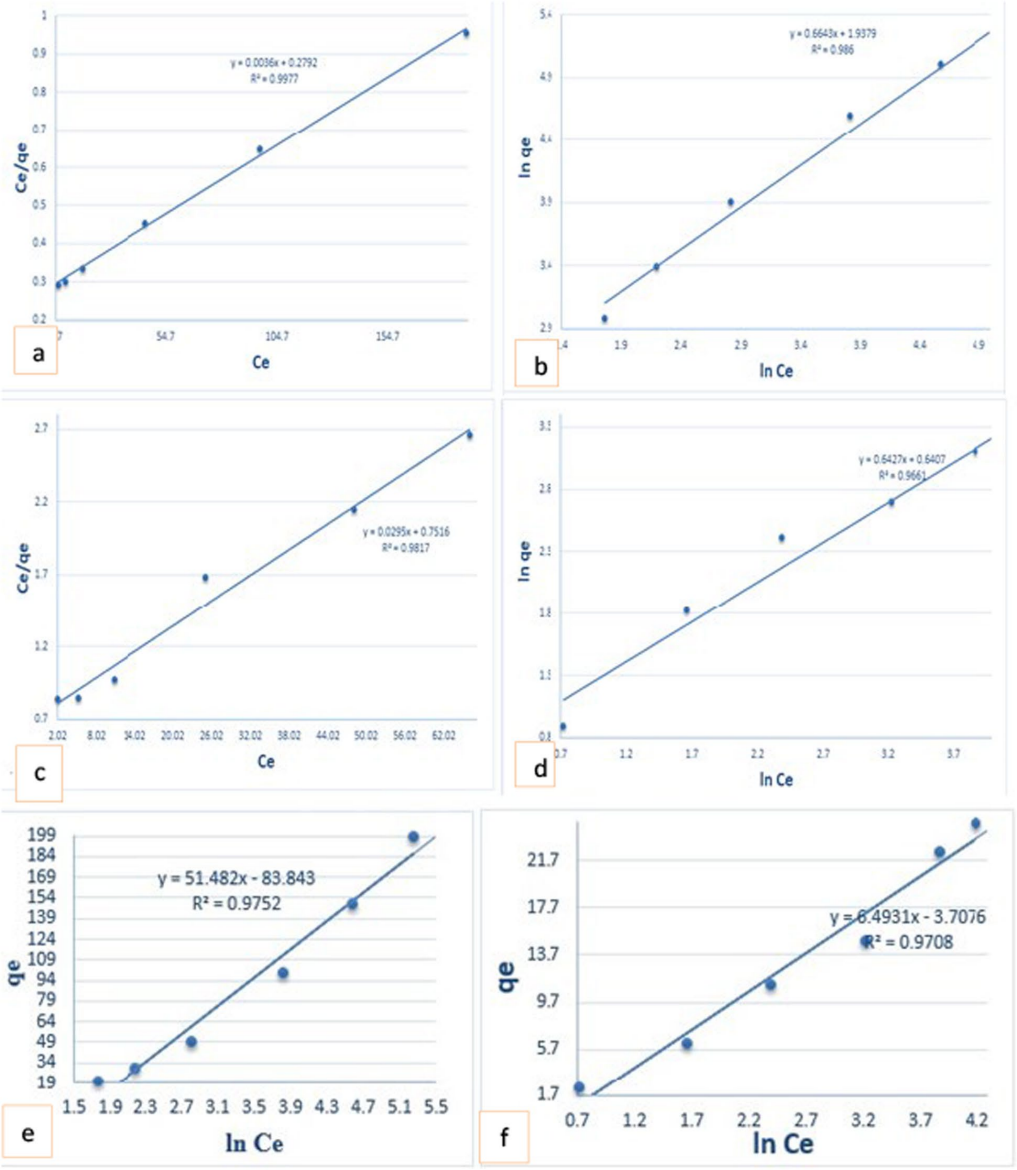
Langmuir (**a**, **b**), Freundlich (**c**, **d**), and Temkin (**e**, **f**) isotherm for MO and Cr(VI) adsorption on GO@Cs-GLA-TiO_2_

The adsorption data were analyzed using Langmuir, Freundlich, Temkin, Dubinin-Radushkevich, and Redlich–Peterson Eqs. (8–12) in Table S4 [69]. The linear plots of these four adsorption models are depicted in Fig. 5, and the corresponding isotherm parameters derived from the slope and intercept in each model are listed in Table 2. Where qe is the amount of metal ion and MO adsorbed on the adsorbent surface (mg g^−1^); K_L_ is the Langmuir constant expressed in (L. mg^−1^), K_T_ is the Temkin constant, and q_max_ is the maximum adsorption capacity when the monolayer is formed on the adsorbent surface.

**Table 2:** Isotherm parameters of MO and Cr(VI) adsorption onto GO@Cs-GLA-TiO_2_ adsorbent

Isotherm	GO@Cs-GLA-TiO_2_	
	MO	Cr(VI)
Langmuir		
qe (mg/g)	277.7	33.8
R^2^	0.997	0.98
R_L_	0.87	0.71
Freundlich		
N	1.5	1.55
K_f_ (L/mg)	7.29	1.89
R^2^	0.986	0.96
Tempkin		
B (J/mol)	40.17	6.4
A_T_ (L/mg)	1.132	62.12
R^2^	0.97	0.97
Dubinin–Radushkevich		
q_m_ Cal(mg/g)	0.313	27.5
β (mol2 kJ^−2^)	0.0126	0.4203
Ea (kJmol^−1^)	6.2	1.09
R^2^	0.998	0.988
Redlich–Peterson		
β	0.004	0.024
aR (L/mg)	0.176	0.49
KRp (L/g)	0.08	0.18
R^2^	0.998	0.99

AT (L/g) is the equilibrium binding constant, KDR (L/g) is the D-R isotherm constant, and related to the mean free energy E (kJ/mol), ε is the polanyi potential, which is equal to R_T_ Ln (1 + 1/Ce) where R (J/mol K) is the gas constant and T (K) is the absolute temperature [53].The Langmuir constant K_L_ and q_m_ values are obtained by plotting a graph of Ce/qe vs Ce (Fig. 5a, b) and the strong R^2^ values (0.997/0.981) for MO and Cr(VI), respectively, indicate that the Langmuir isotherm and the equilibrium data for the adsorption process were closely correlated. The monolayer saturation capacity of MO/Cr(VI) q_max_ was found to be 277.7 ± 1.8 mg g^−1^/33.89 ± 0.48 mg g^−1^ for GO@Cs-GLA-TiO_2_, respectively. The high K_L_ value for GO@Cs-GLA-TiO_2_ suggests a strong MO/Cr(VI) affinity for the latter. The basic feature of the Langmuir isotherm can be characterized by a dimensionless parameter called the separation factor R_L._ The R_L_ values decrease with increasing initial concentration, falling within the range (0 < R_L_ < 1), indicating the suitability of the prepared sorbents for the aqueous removal of MO and Cr(VI). The Freundlich constant ‘n'and Kf’ give an idea about the extent of adsorption and the degree of nonlinearity between the solution concentration and the adsorption process. The Freundlich isotherm (Fig. 5c, d) exhibited a high n value of 1.5 ± 0.01 for MO, and 1.55 ± 0.02 for Cr(VI) for the adsorbent, indicating high favorability of MO and Cr(VI) sorption and suggesting highly heterogeneous surfaces. Conversely, the heterogeneity factor value (1/n < 1) indicates that chemical adsorption is occurring, which supports the previous findings associated with the Lagergren kinetic model. Additionally, the higher K_f_ (mgg^−1^) value for GO@Cs-GLA-TiO_2_ suggests a strong sorption capacity for MO and Cr(VI). Upon comparing the R^2^ values presented in Table 2 for Langmuir models, it can be concluded that both isotherms effectively describe the adsorption process. Furthermore, it has been observed that heterogeneous adsorption is present, extending beyond just monolayer adsorption. This further confirms the heterogeneous nature of the surface of the nanocomposites. As a useful tool for comprehending the interactions between adsorbing species and the adsorbate, the experimental equilibrium data for the adsorption process fit the Temkin model quite well. By analyzing the linear plots of qe against ln Ce (Fig. 5e, f), correlation coefficients (R^2^) ranging from 0.97 to 0.99 were achieved. Table 2 provides further insights, indicating that values of B greater than 1 suggest the occurrence of electrostatic attraction between the heterogeneous surface of the adsorbent and the dye [50].

### Dubinin–Radushkevich isotherm

The Dubinin-Kaganer-Radushkevich (D-K-R) isotherm model is commonly utilized to characterize adsorption processes on surfaces that exhibit both homogeneous and heterogeneous characteristics. This model is particularly effective in estimating the characteristic porosity and the apparent free energy of adsorption, making it a valuable tool in adsorption equilibrium studies. This model is expressed by Eq. (11) in Table 6. According to Table 2, the sorption energy β for GO@Cs-GLA-TiO_2_ was determined to be 0.012 ± 0.008 for (MO), while for Cr(VI), β was reported to be 0.42 ± 0.001. For the GO@Cs-GLA-TiO_2_ composite, the E values for MO and Cr(VI) were found to be 6.2 ± 0.2 and 1.09 ± 0.002 kJ/mol, respectively, indicating that the chemical sorption(MO) and physical sorption(Cr(VI) processes follow an ion-exchange mechanism. It can be concluded that an increase in solution temperature would facilitate the sorption process for MO [68].

### The Redlich–Peterson isotherm

Combining three factors into an empirical equation, the Redlich-Peterson (Rp) model is a cross between the Freundlich and Langmuir isotherms. This paradigm offers a flexible method that works with both homogeneous and heterogeneous systems. Unlike the Langmuir model, the Redlich-Peterson model does not strictly adhere to ideal monolayer adsorption. In its linear form, the (Rp) model can be expressed by Eq. (12 in Table S3), as mentioned by [70]. A and B are the Redlich–Peterson equation constants (L g^−1^ and (L mg^−1^) g, respectively, and g is the exponent having the value between 0 and 1. A is the binding constant (L mg^−1^), and b is the heat of adsorption. The model incorporates the (Rp) constants K_RP_ (Lg^−1^) and αRP (L mg^−1^), along with the exponent β, which falls within the range of 0 to 1. When the exponent β approaches zero, Eq. (9), the Freundlich isotherm will be the preferable isotherm, and when the exponent β approaches 1, Eq. (8), the will be the preferable isotherm [71]. Table 2 displays the heterogeneity (β) values for MO and Cr(VI), which fall within the range of 0.004 ± 0.001 and 0.0244 ± 0.004, respectively, for GO@Cs-GLA-TiO_2_. Table 2 displays the αR (L/g) values for MO 0.017 ± 0.001 and 0.49 ± 0.008 Cr(VI) for the GO@Cs-GLA-TiO_2_ composite, suggesting that the imitation for the Langmuir isotherm was more than the Freundlich. The Redlich–Peterson isotherm model demonstrated the highest R^2^ values for MO 0.998 and Cr(VI) 0.992, when applied to the GO@Cs-GLA-TiO_2_ composites, as indicated in Table 2. The Langmuir and Redlich–Peterson models best fit the experimental data concerning MO and Cr(VI), as these models exhibited R^2^ values approaching unity. Additionally, these values were notably higher compared to those obtained from other isotherm models.

### Thermodynamics study

Methyl orange adsorption onto GO@Cs-GLA-TiO_2_-based biosorbent material was studied thermodynamically to identify critical parameters indicating the viability of the adsorption process, its endothermic or exothermic character, and spontaneity. Accordingly, ΔH and ΔS were found to be 0.207 ± 0.25 kJ/mol and 67.07 ± 1.14 kJ/mol, and 0.011 ± 0.02 JK^−1^/mol and 68.52 ± 1.17 JK^−1^/mol, respectively. These values resulted in negative ΔG values for all temperatures investigated, as presented in Table 3. Furthermore, it was observed that the Gibbs free energy change decreased with increasing temperature, indicating a spontaneous change. Typically, endothermic processes exhibit a positive enthalpy change, and rising temperatures lead to a decrease in the Gibbs free energy change, making the process more favorable. Additionally, the positive ΔS° value obtained suggests the heterogeneity of the adsorption process, supporting the findings of the adsorption isotherm. Overall, the thermodynamic analysis revealed that the adsorption of methyl orange onto GO@Cs-GLA-TiO_2_-based biosorbent material is endothermic, spontaneous, and feasible. Positive ΔH values further confirmed the endothermic nature of adsorption, while the positive ΔS° values indicated a reduction in randomness at the adsorbent-solution interface [73]. Conversely, the negative enthalpy change of Cr(VI) indicates that the adsorption is partially exothermic, and the interactions are predominantly physical [74]. The negative ΔG° value confirmed the feasibility and thermodynamic spontaneity of the adsorption process. However, for MO dye, the ΔG° values decreased with increasing temperature, suggesting that higher temperatures make the adsorption more favorable. In contrast, for Cr(VI), the ΔG° values decreased with increasing temperature, indicating that higher temperatures render the adsorption less favorable. The adsorption capacity of chrome ions onto the adsorbent is influenced by temperature. At high temperatures, the kinetic energy of the system increases, potentially weakening the electrostatic attraction between the adsorbent and chrome ions. This weakening leads to a decrease in the adsorption capacity of the adsorbent for chrome ions. Therefore, temperature plays a significant role in determining the efficiency of the adsorption process [75]. Generally, ΔG° values within the range of (− 20 to 0 kJ/mol) correspond to physical adsorption. In our study, the values ranged from − 20.78 ± 0.4 to − 22.52 ± 0.2 kJ/mol and 20.41 ± 0.57 to 22.13 ± 0.87 kJ/mol, suggesting the physical adsorption of MO/Cr(VI) onto the GO@Cs-GLA-TiO_2_ surface through electrostatic attraction and hydrogen bonding.

**Table 3:** Thermodynamic parameters of MO/Cr(VI) adsorption activity of composites GO@Cs-GLA-TiO_2_

Adsorbent	Temperature, K	(ΔG), kJ mol^−1^	ΔH, kJ mol^−1^	ΔS, kJ mol^−1^	R^2^
GO@Cs-GLA-TiO_2_ MO	298	− 20.781409	0.216821	69.737	0.982
	303	− 21.130094			
	308	− 21.478779			
	318	− 21.827464			
	323	− 22.524834			
GO@Cs-GLA-TiO_2_ Cr(VI)	298	20.4194402	− 0.21267	− 68.5223	0.985
	303	20.7620519			
	308	21.1046635			
	318	21.4472751			
	323	22.1324984			

### Optimization of the response from the Box-Behnken method

**Fig. 6:**
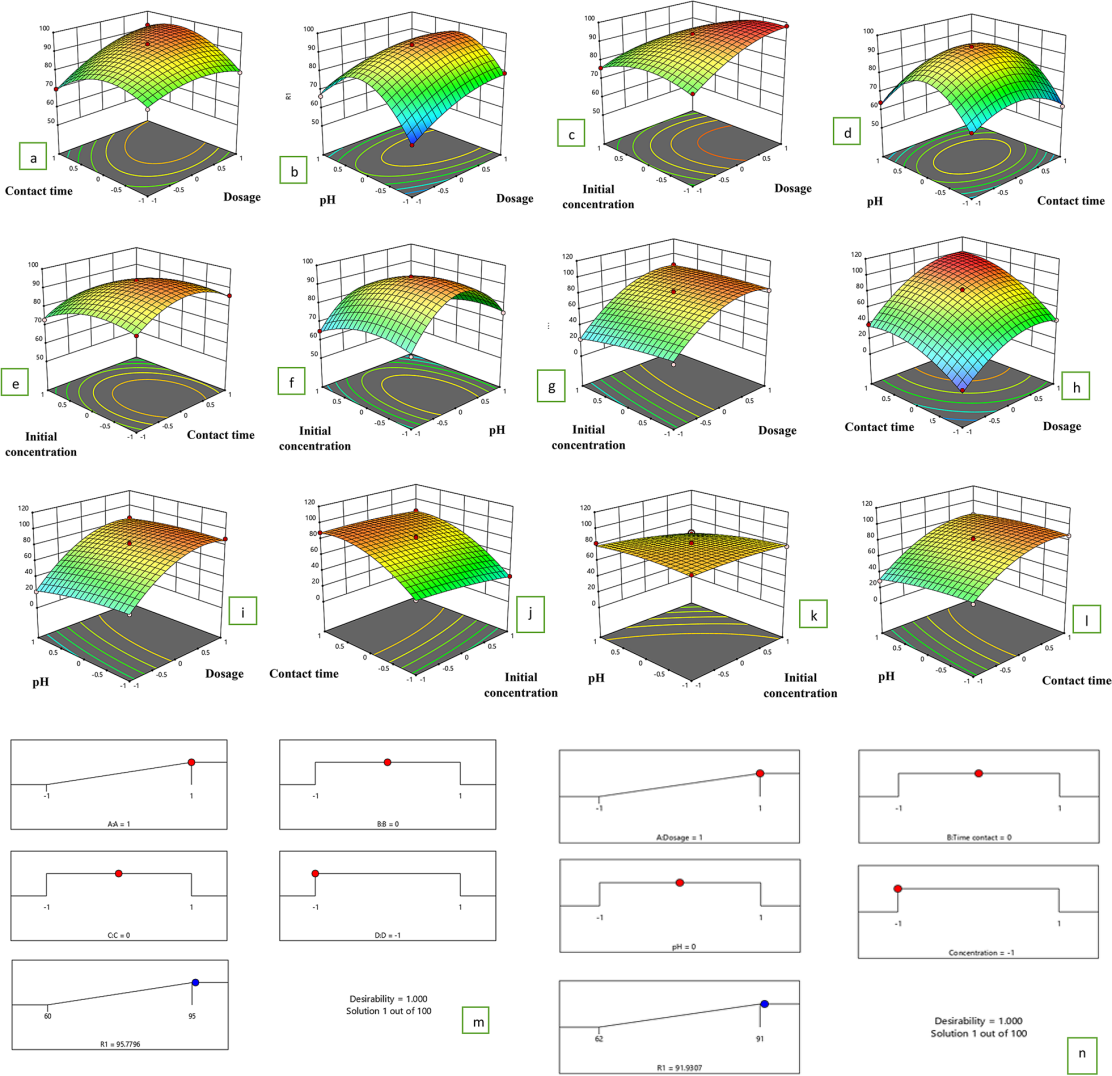
Response surface graph for the removal of MO (%) (**a**, **b**, **c**, **d**, **e**, **f**)/Cr(VI) (**g**, **h**, **I**, **j**, **k**, **l**) by GO@Cs-GLA-TiO_2_ with interactions between (**a**, **g**) contact time and dosage, (**b**, **h**) pH and dosage, and (**c**, **i**) initial concentration of MO/Cr(VI), (**d**, **j**) pH and contact time, and (**e**, **k**) initial concentration of MO/Cr(VI) and contact time, and (**f**, **l**) (**m**, **n**) pH and initial concentration of MO/Cr(VI), and (**o**, **p**) desirability ramps for numerical optimization of four independent variable

The Box-Behnken method was employed to examine the impact of various parameters such as initial dye concentration, dosage, contact time, and pH on the response. Seventeen experiments were conducted, each corresponding to a specific response for every adsorbent, indicating the predicted adsorption capacity. These predictions were derived from second-order polynomial equations fitted using multiple regression analysis, as shown in Eqs. (13, 14).

13 $$\text{Adsorption }(\text{Y})=83.62 + 1.14757 *\text{ A }+ 1.94688 *\text{ B }+ -5.22118 *\text{ C }+ -5.60174 *\text{ D }+ 8.675 *\text{ AB }+ -9.5 *\text{ AC }+ 2.22604 *\text{ AD }+ 2.48646 *\text{ BC }+ -3.42917 *\text{ BD }+ -5.55 *\text{ CD }+ -12.2112 *\text{ A}^2 + 6.8492 *\text{ B}^2 + -5.46226 *\text{ C}^2 + -1.17476 *\text{ D}^2$$

14 $$\text{Adsorption}(\text{Cr}(\text{IV}) =80.78 + 26.6333 *\text{ A }+ -4.86667 *\text{ B }+ 22.6083 *\text{ C }+ -5.15833 *\text{ D }+ 3.975 *\text{ AB }+ 5.2 *\text{ AC }+ 3.275 *\text{ AD }+ 2.825 *\text{ BC }+ -3.95 *\text{ BD }+ 2.95 *\text{ CD }+ -20.0358 *\text{ A}^2 + -2.36083 *\text{ B}^2 + -15.4733 *\text{ C}^2 + -3.39833 *\text{ D}^2$$

where Y, A, B, C, D are the removal efficiency, adsorbent dosage, contact time, pH and the concentration, respectively, of MO/Cr(IV) solution. The adequacy of the model was assessed through an analysis of variance (ANOVA). The findings from the response surface quadratic model fitting in ANOVA are presented in Tables 4 and 5. It was determined that the model is statistically significant, with F-values of 381.3 for MO and 231.2 for Cr(IV). The F-statistical probability value is utilized to evaluate the null hypothesis, indicating that parameters are considered significant if their F-statistical probability value is below 0.05. In this analysis, the significance of model terms such as A, B, C, D, AB, AC, AD, BC, BD, CD, A^2^, B^2^, and C^2^ was established based on their respective probability values [72]. The values of the"Lack of Fit F-value"for MO/Cr(IV) were found to be 0.166 and 0.96, indicating that they are not significant compared to the pure error. This suggests that the model used in the study is applicable and can provide the necessary results. It can be concluded that dose and time have an important role in MO/Cr (VI) adsorption over pH and initial concentration. Higher values of R^2^ and Adj. R^2^ indicated better model fitting. Additionally, a smaller difference between R^2^ and Adj. R^2^ suggested closer agreement between predicted and experimental adsorption capacity is presented in Table 6. All P-values below 0.05 were considered significant for a factor. The overall significance of the model was determined by considering the combination of F and P-values, as well as the signal-to-noise ratio. As the signal-to-noise ratio exceeded 4 for all responses, all models were deemed significant. The dispersion of data points can be determined by calculating the (Std.dev (0.78/2.44)) and the coefficient of variation (CV) (0.99/3.82). A smaller value of CV signifies that the data points are more closely clustered around the mean value, suggesting a higher level of reliability for the model. The reliability of a model can be assessed by evaluating the signal-to-noise ratio (Adequate precision) (AP). In this study, the AP for MO/Cr (VI) was found to be 83.2/52.2, which is greater than 4. This indicates that the model is reliable for predicting experiments. The residual graph is analyzed to confirm whether the model residuals exhibit characteristics of normality, independence, and constancy [33]. Fig. S4a illustrates the normal probability plot of residuals, indicating adherence to a Gaussian distribution and thereby confirming normal distribution characteristics. Fig. S4a displays the chronological plot of residuals, ranging between − 3.93 and 3.93 with an irregular distribution, demonstrating the independence of residuals. According to [73], in a correct and reliable model, residuals show no discernible pattern and are unrelated to other variables. Fig. S4b presents the residuals plotted against predicted values, showing a scattered distribution within a specific range, affirming the constancy of residuals. Smaller residuals indicate closer alignment with the zero line, reflecting a better fit between predicted and experimental values. These analyses collectively validate the reliability of the quadratic polynomial model established by RSM for effectively predicting experimental outcomes. The combined impact of GO@Cs-GLA-TiO_2_ dose and initial MO/Cr(VI) concentration on MO/Cr(VI) removal efficiency is illustrated in Fig. 6a, g. It demonstrates that as the initial MO/Cr(VI) concentration decreases and the GO@Cs-GLA-TiO_2_ dose increases, the removal efficiency of MO/Cr(VI) improves. GO@Cs-GLA-TiO_2_ provides additional surface-active sites, enhancing its capacity to reduce MO/Cr(VI). Higher initial Cr(VI) concentrations lead to more occupied surface-active sites on GO@Cs-GLA-TiO_2_, resulting in lower MO/Cr(VI) removal efficiency [74]. At a GO@Cs-GLA-TiO_2_ dose of 0.4 g/L and an initial Cr(VI) concentration of 15 mg/L, the maximum Cr(VI) removal efficiency reaches 86.3 ± 1.4%. Figure 6b, h depicts the reciprocal effect of contact time and GO@Cs-GLA-TiO_2_ dose on MO/Cr(VI) removal efficiency. Increasing both the GO@Cs-GLA-TiO_2_ dose and contact time enhances the efficiency of MO/Cr(VI) removal. Initially, rapid improvement in Cr(VI) removal efficiency suggests dominant reduction processes. However, prolonged contact times result in a slower increase in MO/Cr(VI) removal efficiency due to the accumulation of Fe(III)–Cr(III) hydroxides hindering the reaction between MO/Cr(VI) and GO@Cs-GLA-TiO_2_ [75]. The curvature of the response surface indicates a significant interaction effect between contact time and GO@Cs-GLA-TiO_2_ dose. A peak removal efficiency of 98.95 ± 0.18% for MO and 96.2 ± 0.13% for Cr(VI) was achieved at a dosage of 1 g/L and a contact time of 90 min. In Fig. 6c, i, the combined effect of GO@Cs-GLA-TiO_2_ of initial Cr(VI) concentration and contact time on MO/Cr(VI) removal shows continuous improvement in removal efficiency with increased contact time and decreased initial MO/Cr(VI) concentration. Extended contact time allows sufficient reaction time for MO/Cr(VI) reduction and adsorption by GO@Cs-GLA-TiO_2_. Figure 6d, j illustrates the interactive influence of GO@Cs-GLA-TiO_2_ dose and initial pH on Cr(VI) removal. Increasing initial pH decreases Cr(VI) removal, whereas increasing GO@Cs-GLA-TiO_2_ dose enhances it. This phenomenon is attributed to the electrostatic properties of GO@Cs-GLA-TiO_2_, which appear negatively charged under alkaline conditions and positively charged under acidic conditions [76]. In alkaline solutions, Cr(VI) predominantly exists as CrO4^2−^, which can repel negatively charged GO@Cs-GLA-TiO_2,_ thus reducing Cr(VI) removal efficiency. A maximum Cr(VI) removal efficiency of 86.3 ± 1.15% is achieved at a GO@Cs-GLA-TiO_2_ dose of 0.4 g/L and an initial pH of 2. Figure 6e, k shows the interactive impact of initial MO/Cr(VI) concentration and initial pH on Cr(VI) removal. Under conditions of high pH and high initial Cr(VI) concentrations, Cr(VI) removal efficiency decreases. The relatively small curvature of the response surface indicates a minor interaction effect between initial Cr(VI) concentration and initial pH on MO/Cr(VI) removal efficiency. At an initial MO concentration of 20 mg/L and an initial pH of 3.7, the maximum MO removal efficiency is 95.3 ± 0.15%. Finally, Fig. 6f, l presents the interaction of contact time and initial pH on MO/Cr(VI) removal. The trend in Cr(VI) removal efficiency follows changes in contact time and opposes variations in initial pH. Similar to the previous cases, contact time and initial pH exert comparable influences on Cr(VI) removal efficiency. A maximum Cr(VI) removal efficiency of 86.8% is observed at a contact time of 90 min and an initial pH of 2.83[77].

**Table 4:** Box–Behnken design-based experimental conditions and results for MO/Cr(VI) adsorption activity of composites GO@Cs-GLA-TiO_2_

Run	A: dose	B: temperature	C: time	D: pH	Response (MO)	Predicted 1	Response (Cr(VI)	Predicted 2
1	0	1	0	− 1	81.77	80.92	86.1	86.10
2	0	0	− 1	− 1	70	67.68	82.9	81.51
3	− 1	0	1	0	60	60.27	22	23.12
4	1	0	0	1	80	80.97	83	81.47
5	0	1	0	1	72	72.12	81.3	81.68
6	− 1	− 1	0	0	77	77.41	61.10	60.78
7	1	− 1	0	0	78.9	77.7	44	44.51
8	1	0	0	− 1	95	95.78	87.9	85.24
9	0	− 1	0	− 1	86	84.62	45.6	47.62
10	0	0	0	0	81	82.2	80	80.78
11	1	0	1	0	72.9	74.15	84.9	83.50
12	0	− 1	0	1	73	74.77	29	31.40
13	0	0	0	0	80	82.2	82.9	80.78
14	0	− 1	− 1	0	66.9	67.24	48	48.24
15	0	1	1	0	68	68.68	84.3	82.89
16	0	1	− 1	0	62	64.02	88	86.97
17	− 1	0	0	1	76	75.88	21	22.49
18	0	− 1	1	0	64.1	64.1	33	32.85
19	− 1	0	0	− 1	78	78.94	39	39.35
20	1	1	0	0	92	90.53	93.2	96.80
21	0	0	0	0	85	82.2	82	80.78
22	0	0	0	0	84	82.2	79	40.80
23	− 1	0	− 1	0	66.4	66.88	87	85.28
24	1	0	− 1	0	79	78.56	84	79.68
25	0	0	1	− 1	75	76.26	78	79.10
26	0	0	− 1	1	70	68.9	82	61.46
27	0	0	1	1	64	62.45	61.3	36.46
28	− 1	1	0	0	70	70.49	78.2	80.78
29	0	0	0	0	81	82.2	80	80.78

**Table 5:** The ANOVA results for the response surface model for the removal of MO dye

Source(b)	Sum of Squares	df	Mean Square	F-value	p-value	
Model	2257.87	14	161.28	54.04	< 0.0001	Significant
A-A	418.90	1	418.90	140.36	< 0.0001	
B-B	7.10	1	7.10	2.38	< 0.0001	
C–C	0.2133	1	0.2133	0.0715	< 0.0001	
D-D	249.07	1	249.07	83.46	< 0.0001	
AB	101.00	1	101.00	33.84	< 0.0001	
AC	44.89	1	44.89	15.04	0.0017	
AD	51.84	1	51.84	17.37	0.0009	
BC	19.36	1	19.36	6.49	0.0233	
BD	4.69	1	4.69	1.57	0.2307	
CD	30.25	1	30.25	10.14	0.0066	
A^2^	0.8194	1	0.8194	0.2746	0.6085	
B^2^	115.01	1	115.01	38.54	< 0.0001	
C^2^	1200.99	1	1200.99	402.42	< 0.0001	
D^2^	0.0046	1	0.0046	0.0015	0.9692	
Residual	41.78	14	2.98			
Lack of Fit	31.78	10	3.18	1.27	0.4406	Not significant
Pure Error	10.00	4	2.50			
Cor Total	2299.65	28				

Std. Dev	0.6445	R^2^	0.9972
Mean	76.54	Adjusted R^2^	0.9943
C.V. %	0.8420	Predicted R^2^	0.9902
		Adeq Precision	29.8520

**Table 6:** Analysis of variance for % of Cr(VI) adsorption activity of composites GO@Cs-GLA-TiO_2_

Source(c)	Sum of Squares	df	Mean Square	F-value	p-value	
Model	18,865.70	14	1347.55	170.52	< 0.0001	Significant
A-dose	8247.76	1	8247.76	1043.67	< 0.0001	
B-Temerature	284.21	1	284.21	35.96	< 0.0001	
C-time	5909.64	1	5909.64	747.80	< 0.0001	
D-ph	319.30	1	319.30	40.40	< 0.0001	
AB	63.20	1	63.20	8.00	0.0134	
AC	62.41	1	62.41	7.90	0.0139	
AD	42.90	1	42.90	5.43	0.0353	
BC	31.92	1	31.92	4.04	0.0641	
BD	62.41	1	62.41	7.90	0.0139	
CD	34.81	1	34.81	4.40	0.0545	
A^2^	2713.33	1	2713.33	343.34	< 0.0001	
B^2^	30.05	1	30.05	3.80	0.0715	
C^2^	1637.79	1	1637.79	207.25	< 0.0001	
D^2^	66.01	1	66.01	8.35	0.0119	
Residual	110.64	14	7.90			
Lack of Fit	100.27	10	10.03	3.87	0.1021	Not significant
Pure Error	10.37	4	2.59			
Cor Total	18,976.34	28				

Std. Dev	2.81	R^2^	0.9942
Mean	63.53	Adjusted R^2^	0.9883
C.V. %	4.42	Predicted R^2^	0.9687
		Adeq Precision	47.8869

### Adaptability function

The desirability function was used to precisely forecast the highest response [78]. This function, ranging from 0 (undesirable) to 1 (desirable), guided the optimization process by assessing various response values. Numerical optimization methods were employed to identify points that maximize the desirability function. As illustrated in Fig. 6m, n, the optimization plot shows the best-predicted values for maximum removal percentages of (MO) and Cr(IV) along with their corresponding desirability function scores. The optimal conditions for achieving the highest MO removal percentage were determined to be an adsorbent dose of 0.6 g, a dye pH of 3, a dye concentration of 20 mg/L, and a contact time of 60 min, resulting in a dye removal efficiency of 95.7%. For Cr(VI), the highest removal percentage was achieved with an adsorbent dose of 0.8g, pH of 2, a concentration of 15 mg/L, and a contact time of 60 min, leading to a removal efficiency of 91.9%.

### Optimization of methyl orange (MO) decolorization using statistical design

At this stage, the most influential variables affecting MO degradation, as identified by RSM, were further fine-tuned using BBD, as shown in Table S5a. Through a 29-run matrix, the concentrations of MO, contact time, pH, and dosage were examined using a four-level BBD design. Table S5a shows the concentrations of the factors that were tested as well as the experimental and anticipated results. Fig. S5a and b demonstrate that the experimental response values for MO decolorization match the normal probability, the studentized residual plot, and the predicted response values.

These predictions were derived from second-order polynomial equations fitted using multiple regression analysis, as shown in Eq. (15).

15 $$\text{Y}= 94.2 + 5.90833 *\text{ A }+ 0.769167 *\text{ B }+ 0.55 *\text{ C }+ -4.9725 *\text{ D }+ 5.025 *\text{ AB }+ -3.35 *\text{ AC }+ -3.6 *\text{ AD }+ 2.2 *\text{ BC }+ -1.0825 *\text{ BD }+ -1.5 *\text{ CD }+ -5.03625 *\text{ A}^2 + -9.6025 *\text{ B}^2 + -19.6237 *\text{ C}^2 + -5.99 *\text{ D}^2$$

Based on the data presented in Table S5 b, the ANOVA results for the model indicate that it is statistically significant. The impressive F value (169.6) and the reduced P values (less than 0.0500) for (MO) indicate the significance of the model terms. The variables A, D, AB, AC, AD, BC, CD, A^2^, B^2^, C^2^, and D^2^ were identified as significant model terms for decolorization based on their respective P values. Additionally, the ANOVA results in Table S5 b reveal that the linear effects of dye temperature, pH, and concentration played an increasingly important role in MO dye decolorization. The lack of fit, with an F value of 0.395, was found to be insignificant in terms of pure error. The predicted R^2^ for immobilized *Trichoderma sp*. was 0.971, which aligns well with the adjusted R^2^ of 0.988. These results suggest that the developed model was satisfactory. The ratio of (AP) 42.2 for *Trichoderma sp*. was notably high, suggesting that the experimental data is reliable. Additionally, as shown in Table S5c, the coefficient of variation (CV%) values for *Trichoderma sp*. were recorded at 1.61. This low CV% indicates minimal deviations between the experimental and predicted values, reflecting a high level of consistency in the results. Fig. S5c displays a Box-Cox diagram illustrating the model's changes in MO removal (%) using *Trichoderma sp*. composite, as determined by a quadratic polynomial. The optimal lambda value (λ = 0.299) suggests that data transformation is not required. The red line indicates the minimum (−0.600) and maximum (1.16) values, along with the lambdas at the 95% confidence interval, as depicted in Fig. S5e, f.

### Interactive effect of pH on the decolorization of MO dye

The impact of different pH levels on the biosorption of MO by the fungus was investigated in the pH range of 3–9. The results showed that the highest degree of decolorization, reaching 95.6%, was achieved at a pH of 7.0. However, at a pH of 9, the decolorization rate decreased to 44.6%. Fig. S6b, f demonstrates the decreasing efficacy of dye decolorization as the pH level increases. Additionally, Fig. S6d illustrates that the removal efficiency was 87.7 ± 1.05% at a pH of 7.0 and a temperature of 30 °C, but it decreased to 69.6% at a pH of 5 and the same temperature. These findings align with the research conducted by [79], who discovered that the efficiency of MO decolorization using *Stenotrophomonas acidaminiphila* was approximately 82 ± 1.04% at a pH of 7. In a study conducted by [80], it was observed that the highest degradation of MO dye (89%) by *Aeromonas hydrophila* occurred at pH 7. [23], who discovered that the efficiency of Congo red decolorization using *A. flavus* was approximately 93 ± 0.17% at a pH of 4. pH plays a crucial role in affecting microbial growth, enzymatic functions, and the efficiency of biodegradation processes. Elevated pH levels can hinder decolorization by reducing enzymatic activity and overall bioactivity. Conversely, lower pH levels, characterized by the presence of H^+^ ions, can impede the action of dye cations, resulting in decreased decolorization efficiency [81].

### The interactive effect of temperature on the decolorization of MO dye

Various environmental factors played a crucial role in the degradation of MO using immobilized *Trichoderma sp*. This particular fungal strain exhibited efficient degradation of MO within the temperature range of 303 °C to 318 °C. The graphical representation in Fig. S6a and e indicates that the rate of MO decolorization increased with an increase in temperature from 25 °C to 45 °C. Moreover, immobilized *Trichoderma sp* achieved a maximum decolorization efficiency of 95.24 ± 1.05% at 303 °C. However, as the temperature further rose to 318 °C, the decolorization activity decreased significantly by 52.6% due to the loss of cell viability or inactivation of the decolorizing enzymes [82]. These findings align with the observations made by [35], the ideal temperature for attaining 92% decolorization of malachite green dye using *Mucor sp*. on an activated carbon composite was found to be 30 °C.

### The interactive effect of concentration on the decolorization of MO dye

The adsorption behavior of (MO) was investigated in a concentration range of 10–200 mg/L at a pH of 7.0. The immobilized fungus exhibited a remarkable elimination rate of 43.7–97.4 ± 0.16% for MO concentrations ranging from 10 to 200 mg/L. However, the decolorization efficiency of immobilized *Trichoderma sp.* fell below 43.7% as the initial concentration of MO approached 200 mg/L. These findings indicate that high concentrations of MO hinder the growth and development of immobilized *Trichoderma* sp. Fig. S6c and e. At 200 mg/L of MO, the immobilized fungus experienced suppression due to the presence of sulfonic acid on the aromatic ring formed in the medium as a result of the increased concentration of MO. This sulfonic acid inhibited nucleic acid synthesis and microbial cell proliferation, leading to a decrease in the effectiveness of the immobilized fungus [83]. These findings align with a study conducted by [84], who observed that immobilized *Aspergillus fumigatus* in polyurethane foam achieved an optimal malachite green decolorization percentage of approximately 97.52 ± 0.15% at 40 mg/L, which decreased to 23 ± 1.15% at 70 mg/L. The impact of temperature (ranging from 25 to 45 °C) on the decolorization of MO dye was investigated using a temperature-controlled incubator (L9-VODS-10, Velp, Italy). The results showed that the efficiency of MO dye decolorization decreased both below and above 30 °C. Minor decreases in decolorization efficiency were observed at 20 and 45 °C. The highest decolorization percentage (97%) was achieved at 30 °C, while at 20 °C and 35 °C, decolorization rates of 32.5% and 89.05% were observed, respectively. These findings align with previous studies. Kishor et al. [85] also observed a comparable trend in MO dye decolorization at 30 °C. Hussien Hamad, [35] reported that the optimal decolorization of MG dye occurred at 30 °C. Similarly [80], observed a comparable trend in the decolorization of MO dye at 30 °C. Consequently, the reduced decolorization efficiency at lower temperatures could be linked to a decline in microbial growth and enzymatic activity. On the other hand, at higher temperatures, enzyme activity decreases due to enzyme denaturation [86].

### Effect of contact time on the decolorization of MO dye

At the optimal concentration of the dye (50 mg/L) and the dosage of the biosorbent (6 g/L), we investigated the effect of contact time on the removal of the MO dye over a period of 3 to 9 days. Fig. S6a, b and d depict the impact of contact time on the elimination of the MO dye. The adsorption efficiency of MO ranged from 3 to 9 days, resulting in removal efficiencies of 72% and 97.78%, respectively. Based on the data, we determined that the equilibrium time in the sorption process was 7 days, as no further improvement in adsorption was observed beyond this point. Therefore, the decolorization of MO dye was tested at various concentrations, ranging from 5 to 200 mg/L. The results indicated that the decolorization efficiency of MO dye by biosorbent decreased with an increase in dye concentration, as illustrated in Fig. S6c. At dye concentrations of 5–50 mg/L, decolorization percentages ranged from 96.2 ± 0.12% to 98.9 ± 0.14%. For concentrations of 100–150 mg/L, decolorization percentages ranged from 56.3% to 39.8%. Only 13% decolorization was achieved at a dye concentration of 200 mg/L. This decrease in decolorization efficiency at higher dye concentrations can be attributed to the inhibition of microbial growth and metabolic activity of bacteria [16]. The large surface area accessible for dye adsorption in the early phases is responsible for the initial high removal effectiveness at 7 days of contact time. But as time went on, the adsorbent’s capacity steadily decreased because the solute molecules’ repulsive effects on the solid and bulk phases made it harder to occupy the remaining empty surface sites [87]. These findings align with the observations made by [88], who reported that *Aeromonas hydrophila*. Was able to decolorize 89% of MO within 5 days. Similar findings have been reported in studies conducted by [89] on the decolorization of Triphenylmethane by *Ganoderma resinaceum*. The study found that *Ganoderma resinaceum* was able to achieve a high percentage of decolorization of Triphenylmethane over an 8-day period.

### Optimization of experimental conditions and validation of the polynomial models

The optimal conditions for MO adsorption by immobilized fungal biomass, determined from Fig. S6d, were set as a temperature of 30 °C, a pH of 7, a dye concentration of 10 mg/L, and a contact time of 3 days. To assess the accuracy of this optimization, the experimental results were compared with the predicted values. Remarkably, there was a close match between the observed (97.78 ± 0.11%) and predicted (97.9 ± 0.11%) values, underscoring the precision and reliability of the model. This strong correlation between experimental and predicted outcomes confirmed the reliability of the modeling approach using RSM.

### Optimization of the ANN structure

**Fig. 7:**
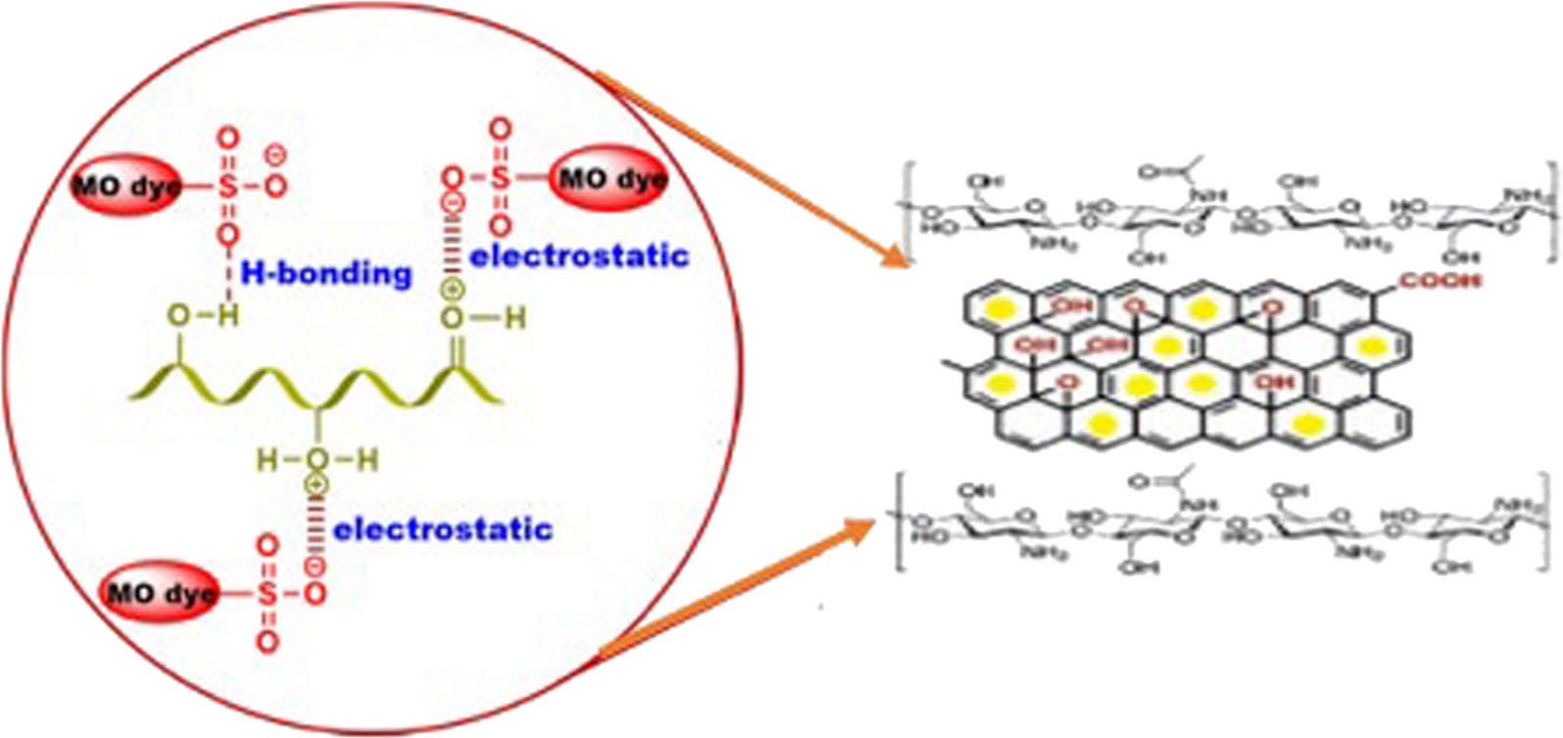
Adsorption mechanisms of MO by GO@Cs-GLA-TiO_2_ hybrid

Artificial Neural Network (ANN) models have demonstrated significant utility in accurately analyzing datasets with minimal error [90].To identify the most effective topology for modeling the MO degradation process, various configurations were tested, varying the hidden layer neuron count from 2 to 20. The study investigated the correlation between the number of neurons in the hidden layer and the network error through mean square error (MSE) calculations [91]. The research employed response surface methodology (RSM) data to optimize the ANN design matrix and determine the best parameter combination. The dataset, obtained from Box-Behnken Design (BBD) optimization comprising 29 points, was partitioned into training, validation, and testing subsets for ANN prediction. Following MATLAB's neural network toolbox guidelines, 70 ± 1.0% of the dataset was allocated for training, with 15% each for validation and testing, ensuring robustness against overfitting [92]. Specifically, 19 datasets were used for training, 4 for testing, and 4 for validation. MSE and R^2^ values were computed across different hidden layer neuron configurations to optimize the ANN model. The ANN model's performance improved with increasing neurons in the hidden layer, resulting in lower MSE, reflecting enhanced model accuracy [93]. Ultimately, a three-layer architecture with a 4–5-1 topology was identified as optimal, achieving an MSE of 4.06 and an R^2^ value of 0.983. Parameter settings were fine-tuned based on minimizing MSE across all datasets [92]. Regression analysis was conducted to evaluate the correlation between ANN outputs and targets. Fig. S7 illustrates the plot of network outputs versus targets as small open circles. The plot depicted in Fig. S7a corresponds to the ANN architecture with a 4–5-1 topology, showcasing correlation coefficients of 0.999 (training), 0.999 (validation), and 0.999 (testing). These high R^2^ values indicate a strong correlation between predicted and actual outcomes, highlighting excellent regression performance [93]. Further evaluation of model performance included the construction of an error histogram post-feedforward neural network training, as illustrated in Fig. S7b. This histogram categorizes errors into bins, demonstrating the distribution of prediction accuracy relative to target values. Notably, the histogram peak at an error value of 5.9e-^07^ indicates a significant portion of the dataset exhibits errors around this magnitude. Figure 7c presents the MSE graph across ten epochs for training, testing, and validation datasets. Optimal validation performance, approximately 0.025, was achieved during the 1000th epoch, specifically for MO degradation facilitated by fungal @Cs GLA-GO. To assess precision, the ANN model's forecasted outcomes were compared against empirical data, visualized in Fig. S7d. The model demonstrated prediction errors ranging from a maximum of 0.95% to a minimum of 0.35%, showing robust alignment with experimental findings. The ANN analysis also investigated variable importance, focusing on fungal @Cs-GA-GO biosorbent dose (A), pH (B), contact time (C), and initial MO concentration (D). Dosage, pH, and time emerged as the most influential factors, with dosage followed by time and pH playing a comparatively minor role in MO removal efficiency. These findings corroborate RSM analyses, which similarly ranked initial concentration as the least impactful variable. The sensitivity analysis underscored the relative impacts of these factors, supporting strategies to enhance MO adsorption efficiency by increasing contact time and biosorbent dose, reducing initial concentration, and maintaining pH near 7. This integrated approach aligns with both quadratic equations from RSM and ANN models, offering actionable insights for optimizing MO removal efficiency.

### Comparison of RSM and ANN predictions on the removal efficiency of the MO dye

The study employed both RSM and ANN to predict the removal efficiencies of MO. The R^2^ values obtained for the RSM and ANN models were 0.998 and 0.999, respectively. These values indicate that both models accurately predicted the removal rates of MO. However, the RSM model had a smaller standard error (SE) of 0.0205 compared to the ANN model’s SE of 0.14, suggesting that the RSM model had a slightly better prediction performance in terms of deviation from actual responses. It is worth noting that the RSM and ANN models demonstrated superior fitting to the experimental data, as evidenced by their higher R^2^ values. Table S6 provides a summary of the experimental percentage removals alongside their predicted values from both RSM and ANN. The comparison of the predicted values with the experimental data further supports the accuracy of both models. The study also conducted a sensitivity analysis to determine the relative importance of different variables in relation to the performance of the ANN system. The analysis revealed that the adsorbent dose, contact time, and pH had the most significant influence on the initial MO concentration. In conclusion, both the RSM and ANN models demonstrated their capability to accurately predict the removal efficiencies of MO. While the RSM model had a slightly better prediction performance, both models provided valuable insights for optimizing MO adsorption removal efficiency [94].

### Biodegradation kinetics of methyl orange (MO)

In the study, the researchers investigated the effect of MO concentration on microbial development. They found that the length of the lag phase, represented as *t*^0^​, increased exponentially with increasing MO concentration within the range of 10–200 mg L^−1^ (Fig. S8). This suggests that MO has an inhibiting effect on microbial growth, particularly at higher concentrations. These findings align with previous studies conducted on mixed cultures [35].

### Relationship between lag phase and maximum specific growth rate

To gain further insight into the impact of the lag phase, the researchers compared its evolution with the maximum specific growth rate. They observed two distinct trends based on the MO concentration: one below 50 mg L^−1^ and one above 50 mg L^−1^ (Fig. S8a). For concentrations below 50 mg L^−1^, the length of the lag phase, *t*^0^​, exhibited a linear increase with an increase in the maximum specific growth rate. This indicates that at lower MO concentrations, a higher growth rate is associated with a longer lag phase. However, for concentrations above 50 mg L^−1^, a contrary trend was observed (Fig. S8b). Here, the length of the lag phase, *t*^0^​, increased as the maximum specific growth rate decreased. This suggests that at higher MO concentrations, the lag phase becomes longer despite a decrease in the growth rate. To further analyze the data, the researchers attempted to fit curves using models such as Monod, Luong, Aiba–Edward, Han, and Levenspiel (Fig. S9a, b, c, d, e). In the study, the researchers aimed to determine the most accurate kinetic model for their analysis. They evaluated four different models: Luong, Han, Levenspiel, Aiba-Edward, Monod, and Haldane. The accuracy and statistical analyses of these models were conducted to assess their performance. According to the results, the Han and Levenspiel models provided reasonably acceptable results based on the software output and visual examination. However, the Haldane model emerged as the most accurate model among the evaluated models, as it had the minimum root-mean-square error (RMSE) and AICc values, and the maximum adjusted R^2^. The Bf​ values for the Haldane model were significant and near 1.0, further validating its superiority. Furthermore, an F-test was conducted to compare the Haldane model with the other models. The results indicated that the Haldane model outperformed the Aiba-Edward, Han, Levenspiel, Luong, and Monod models with percentages of 0.956%, 0.854%, 0.913%, 0.527%, and 0.53%, respectively. This confirms that the Haldane model was the most suitable choice for the analysis. The computed values for the Han and Levenspiel constants in this study, as presented in Table S6, are as follows: the inhibition constant rate symbolized by the maximal growth rate (umax​) was 0.5 h^−1^, the half-saturation constant (Ks​) was 50 mg L^−1^, and the inhibition constant (Ki​) was also 100 mg L^−1^.

### Comparative analysis of GO@Cs-GLA-TiO_2_ adsorption capacity against various adsorbents

Table S7 presents a comparative analysis of the maximum sorption capacities of different adsorbents for MO and Cr(VI) as reported in previous studies. The adsorption capacities exhibited by the GO@Cs-GLA-TiO_2_ composite surpass those of other reported adsorbents. The characterization findings of the GO@Cs-GLA-TiO_2_ composite reveal the presence of numerous binding groups and a substantial surface area, which are conducive to the adsorption of MO and Cr(VI), thus significantly enhancing the removal process.

### Adsorption mechanisms

The removal of dye using GO@Cs-GLA-TiO_2_ involves a variety of mechanisms. According to the literature, surface functional groups on GO@Cs-GLA-TiO_2_ nano-adsorbents play a critical role in dye adsorption. Key functional groups such as -OH, -NH_2_, -NH, -CO, C = C, -CH, -COOH, and Fe-O_3_ are identified through FT-IR, XRD, SEM, and EDX analyses as primarily involved in MO dye adsorption on GO@Cs-GLA-TiO_2_. EDAX spectra show new peaks corresponding to sulfur (S) and chromium (Cr) in MO- and Cr(VI)-adsorbed GO@Cs-GLA-TiO_2_ composites, confirming successful sorption (Fig. 7). FTIR analysis of the adsorbent post-dye adsorption indicates shifts in -OH or -NH_2_ peaks, highlighting hydrogen bonding between MO -OH and -NH_2_ groups and the GO@Cs-GLA-TiO_2_ adsorbent. Additionally, electrostatic attraction may occur between the positively charged GO@Cs-GLA-TiO_2_ surface and the negatively charged -SO_3_- groups of MO. Intra-particle diffusion is also noted, facilitating dye adsorption on GO@Cs-GLA-TiO_2_ surfaces [5]. The presence of sulfonated groups on anionic dyes confirms chemisorption, attracted to the nanoparticles'positive surface charge. In summary, GO@Cs-GLA-TiO_2_ adsorbents facilitate MO adsorption through mechanisms such as hydrogen bonding, electrostatic attraction, and π-π stacking interactions with its aromatic structure [80]. Cr(VI) ions are adsorbed through electrostatic forces and surface complexation with positively charged GO@Cs-GLA-TiO_2_[95]. The pH of the zero-point charge (pHzpc) of the adsorbent is 6.2. The positively charged reactive sites of the GO@Cs-GLA-TiO_2_ hybrids readily interact with negatively charged pollutants through various mechanisms, including electrostatic force of attraction, π-π stacking interactions, and surface complexation. These interactions play a significant role in the adsorption of pollutants such as MO dye and Cr(VI) ions onto the GO@Cs-GLA-TiO_2_ hybrids. It is important to note that the adsorption efficiency of the GO@Cs-GLA-TiO_2_ hybrids decreases under basic conditions (pH higher than pHzpc) due to electrostatic repulsion between the negatively charged surface sites of the hybrids and the MO dye and Cr(VI) ions[77].

### Reusability of GO @Cs-GLA-TiO_2_ composite

**Fig. 8:**
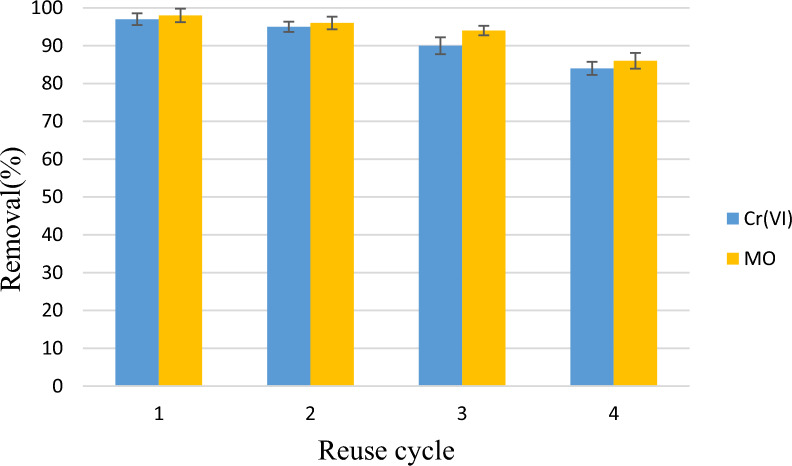
Repeatable reuse cycles of the used adsorbent GO@Cs-GLA-TiO_2_ for the removal of MO and Cr(VI)

Figure 8 showcases the impressive reusability of the GO@Cs-GLA-TiO_2_ composite. After undergoing four adsorption/desorption cycles, the composite demonstrated substantial regeneration capabilities, maintaining removal efficiencies of over 85 ± 4.6% for Cr(VI) and 88.13 ± 3.05% for MO. This indicates that the GO@Cs-GLA-TiO_2_ composite can be utilized repeatedly without significant deterioration in its efficacy, highlighting its stability. Such resilience positions the GO@Cs-GLA-TiO_2_ composite as a promising candidate for industrial adsorption processes. The evaluation of an adsorbent’s recycling and stability is crucial when considering its application in wastewater treatment. In the case of GO@Cs-GLA-TiO_2_, four cycles of Cr(VI) and MO adsorption were conducted, followed by regeneration using a 4% NaCl solution in a water/ethanol mixture. The regenerated nanocomposite consistently displayed efficient adsorption across cycles, indicating the mechanical stability of the nanocomposite. Moreover, the repeated use of the nanocomposite increased roughness, implying an expansion of the specific surface area and further supporting its efficacy. As a result, the developed nanocomposite proves to be a promising solution for the efficient recovery of cationic dyes from contaminated water sources. The assessment of the recycling and stability of the GO@Cs-GLA-TiO_2_ demonstrates its potential for sustainable and effective wastewater treatment.

### Cost analysis

A cost evaluation study was conducted to assess the economic viability of sustainable nanocomposites for water remediation, as shown in Table S8. This cost–benefit analysis underscores the sustainable and cost-effective nature of these nanomaterials, highlighting their potential for practical applications in MO remediation from aqueous media [96]. Comparatively, the total production cost per gram in this study was found to be more economical than that reported by [97], who synthesized phengite clay at US$2.629/g for treating Congo red.

## Conclusions

In summary, the GO@Cs–GLA–TiO₂ composite was successfully synthesized for the removal of methyl orange (MO) and Cr(VI) ions from aqueous solutions. Analytical techniques such as XRD, FTIR, and SEM confirmed the successful formation of the composite material. The adsorption behavior of the Langmuir isotherm model in single-component systems reveals maximum adsorption capacities of 277.7 ± 1.8 mg g⁻^1^ for methyl orange and 33.3 ± 0.48 mg g⁻^1^ for chromium(VI) (Cr(VI). This model indicates that the adsorption mechanism involves monolayer adsorption on a homogeneous surface. Additionally, the pseudo-second-order model fits well for both MO and Cr(VI), with equilibrium adsorption capacities (qe_cal_) of 20.04 ± 0.06 mg/g for MO and 2.54 ± 0.015 mg/g for Cr(VI). The thermodynamic analysis revealed that the adsorption of MO is endothermic and spontaneous, whereas the adsorption of Cr(VI) is exothermic and spontaneous. The optimization plot shows the best-predicted values for maximum removal percentages of (MO) and Cr(IV) along with their corresponding desirability function scores. The optimal conditions for achieving the highest MO removal percentage were determined to be an adsorbent dose of 0.6 g, a dye pH of 3, a dye concentration of 20 mg/L, and a contact time of 60 min, resulting in a dye removal efficiency of 95.7%. For Cr(VI), the highest removal percentage was achieved with an adsorbent dose of 0.8 g, a pH of 2, a concentration of 15 mg/L, and a contact time of 60 min, leading to a removal efficiency of 91.9%. Optimization of methyl orange (MO) decolorization using statistical design. These findings indicate that the adsorption of both contaminants is thermodynamically favorable, with distinct energetic characteristics influencing their adsorption behavior. To optimize the process, both RSM and ANN were employed. Both models accurately predicted MO removal efficiency, with RSM showing slightly better performance (R^2^ = 0.998) compared to ANN (R^2^ = 0.999). Regeneration studies demonstrated that the GO@Cs–GLA–TiO₂ composite retained excellent performance over five consecutive adsorption and desorption cycles, with minimal loss in efficiency. After four cycles, the composite maintained 85 ± 4.6% of its Cr(VI) removal capacity and 88.13 ± 3.05% of its MO removal capacity, underscoring its economic viability and long-term reusability. Cost analysis revealed a low production cost of approximately $0.123 g, which can be further reduced through reuse, supporting the composite’s economic and environmental sustainability. Additionally, the biodegradation of MO was best described by the Haldane model, which provided the most accurate fit among the tested kinetic models, indicating its potential for modeling and optimizing the biodegradation process. This study provides significant insight into the development of an effective, selective, and reusable adsorbent for the removal of both organic and inorganic pollutants from water. Its high adsorption capacity, regeneration potential, and cost-effectiveness make GO@Cs–GLA–TiO₂ a promising candidate for practical water treatment applications.

## Supplementary Information

Supplementary material 1

## Data Availability

The sequence data for Trichoderma viride isolate MT, including the small subunit ribosomal RNA gene (partial), internal transcribed spacer 1, 5.8S ribosomal RNA gene, internal transcribed spacer 2 (complete), and large subunit ribosomal RNA gene (partial), have been deposited in GenBank under the accession number PQ462654.1 and are publicly available at: https://www.ncbi.nlm.nih.gov/nuccore/PQ462654.1.
