# Supplementary material for: A novel hybrid approach for predicting and optimizing the adsorption of methyl orange and Cr(VI) removal from aqueous solutions using fungal-cross linked chitosan integrated into graphene oxide as a cost-effective adsorbent
Source: BMC Chem. 2025 Jul 3;19(1):193. doi: 10.1186/s13065-025-01542-x (PMC12225185; doi:10.1186/s13065-025-01542-x)
Supplement: Supplementary file 1 — Supplementary material 1 [file 13065_2025_1542_MOESM1_ESM.docx]

**Supplementary material**

**A novel hybrid approach for predicting and optimizing the adsorption of methyl orange and Cr(VI) removal from aqueous solutions using fungal-cross linked chitosan integrated into graphene oxide as a cost-effective adsorbent**

**Mohammed T.M. H.Hamad**

Central Laboratory for Environmental Quality Monitoring, National Water Research Center, Egypt

**Corresponding author:**

Mohammed T.M.H.Hamad

**e-mail:** [mohamed_taha@nwrc.gov.eg](mailto:mohamed_taha@nwrc.gov.eg)

**Contents**

Fig. S1 Phylogenetic tree of the fungal isolate *Trichoderma sp*

Fig.S2. FTIR spectra of (a)Cs; (b) GO @Cs‑GLA-TiO_2_ composite; (c) TiO_2_; (d) GO @Cs‑GLA-TiO_2_ composite after dye (e ) MO and(f) Cr(VI).

Fig. S3. (a-b-c-d) the XRD images of GO, TiO_2_,CS, and GO @Cs‑GLA-TiO_2_ composite

Fig.S4 (a)The actual vs predicted removal percentage, (b) The normal probability vs the plot of the studentized residuals, (c),(d) The cook’s distance for each of the experimental runs, Box–Cox plot (e,f) each of the variables for the removal of MO and Cr(VI) adsorption on GO@Cs-GLA-TiO_2_.

Fig.S5 (a)The actual vs predicted removal percentage, (b) The normal probability vs the plot of the studentized residuals, (c),(d) The cook’s distance for each of the experimental runs, each of the variables for the removal of MO adsorption on fungal@Cs-GLA-GO.

Fig. S6 Response surface graph for the removal of MO (%) by biosorbent with interactions between(a) temperature and dosage, (b) pH and dosage, and (c) initial concentration of MO (d) pH and temperature, and(e) initial concentration of MO and temperature, and (fl) pH and initial concentration of MO, and (m) desirability ramps for numerical optimization of four independent variable.

Fig. S7. RSM-ANN model: (a) Linear regression for the ANN-MO model training, verification, testing, and combined input data sets, (b) error histogram, and (c) MSE plot for training, validation, testing, and all data and(d) The percentage of the prediction error of the MO-ANN model.

Fig. S8 Time of the lag phase (t0) versus the initial MO concentration (a), and (b) (S_0_) maximum specific growth rate μm versus the time of the lag phase t_0_ for S_0_ below

Fig. S9 Experimental and predicted speciﬁc substrate consumption rates at diﬀerent MO concentrations due to diﬀerent models.

Table S1(a) Coded levels and design range of variables.

Table S2. Characterization methods and analyzed properties

Table S3 Kinetic models and their linearized expressions in the adsorption of MO/Cr(VI)by GO@Cs-GLA-TiO_2_.

Table S4 Isotherm models and their linearized expressions in the adsorption of MO and Cr(VI) onto GO@Cs-GLA-TiO_2_ adsorbent.

Table S5(a) Coded levels and design range of variables.

Table S5 (b)The ANOVA results for the response surface model for the removal of MO dye.

Table S5c Box–Behnken design-based experimental conditions and results for MO adsorption activity of composites fungal @fungal@Cs-GLA-GO_._

Table S6 Various kinetic models for effect of substrate on growth rate.

Table S7 Comparative study for different biomass based adsorbents applied for methyl orange adsorption.

Table S8 Cost analysis of materials used for adsorption using GO @Cs‑GLA-TiO_2_.


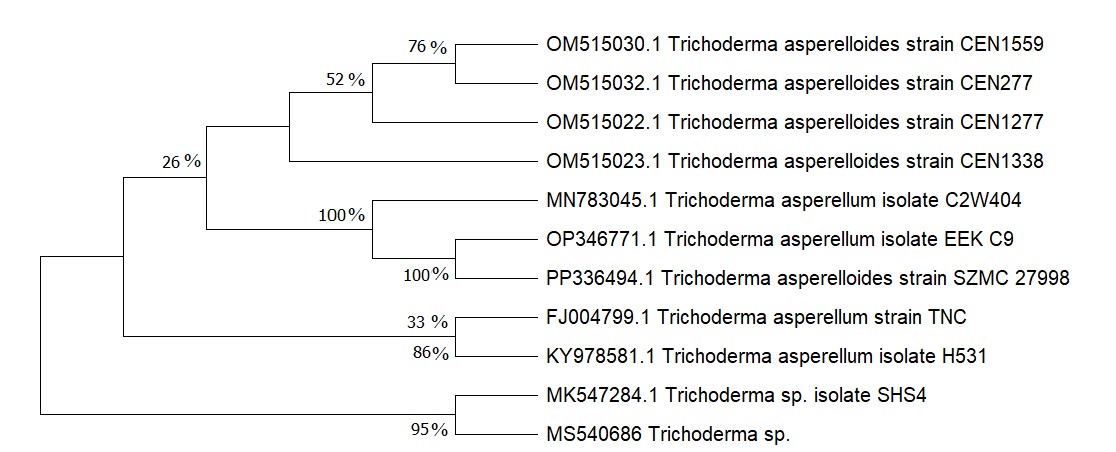


Fig. S1 Phylogenetic tree of the fungal isolate *Trichoderma sp*


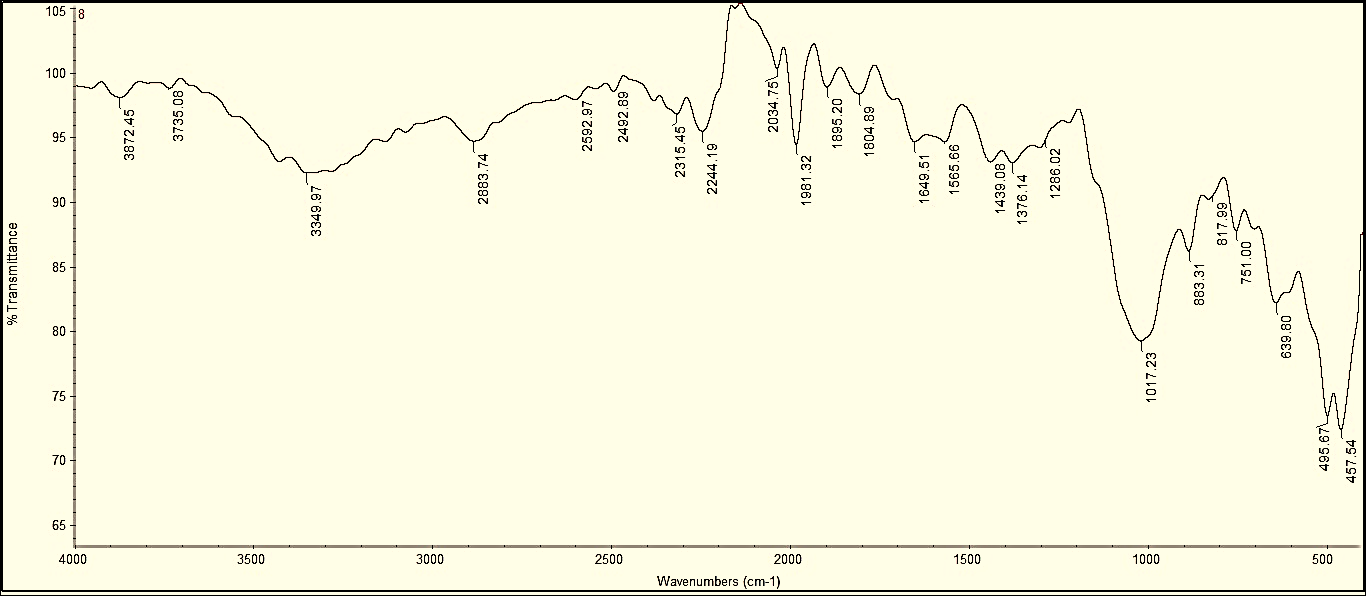


a


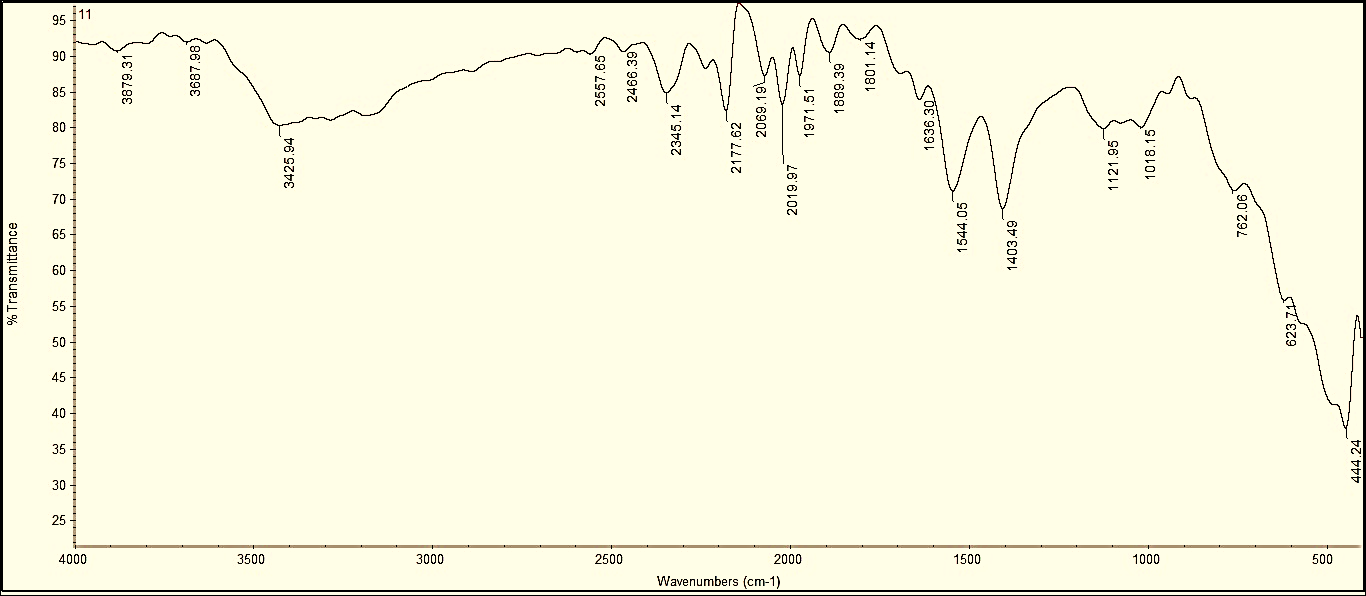


b


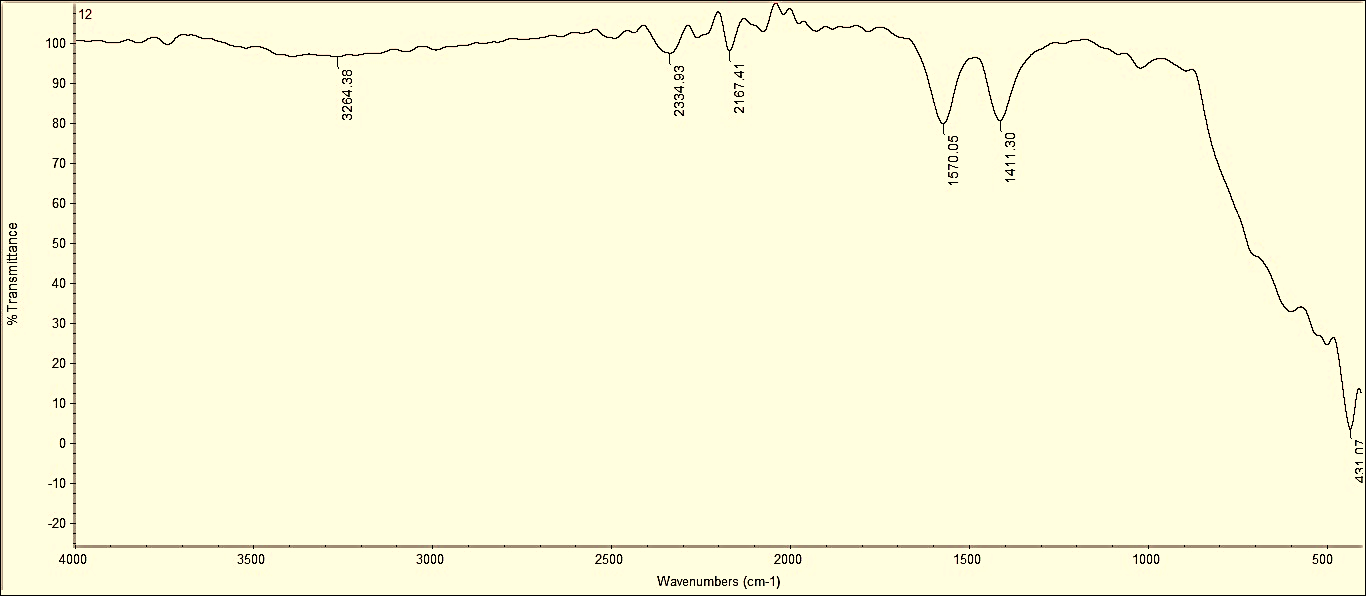


c


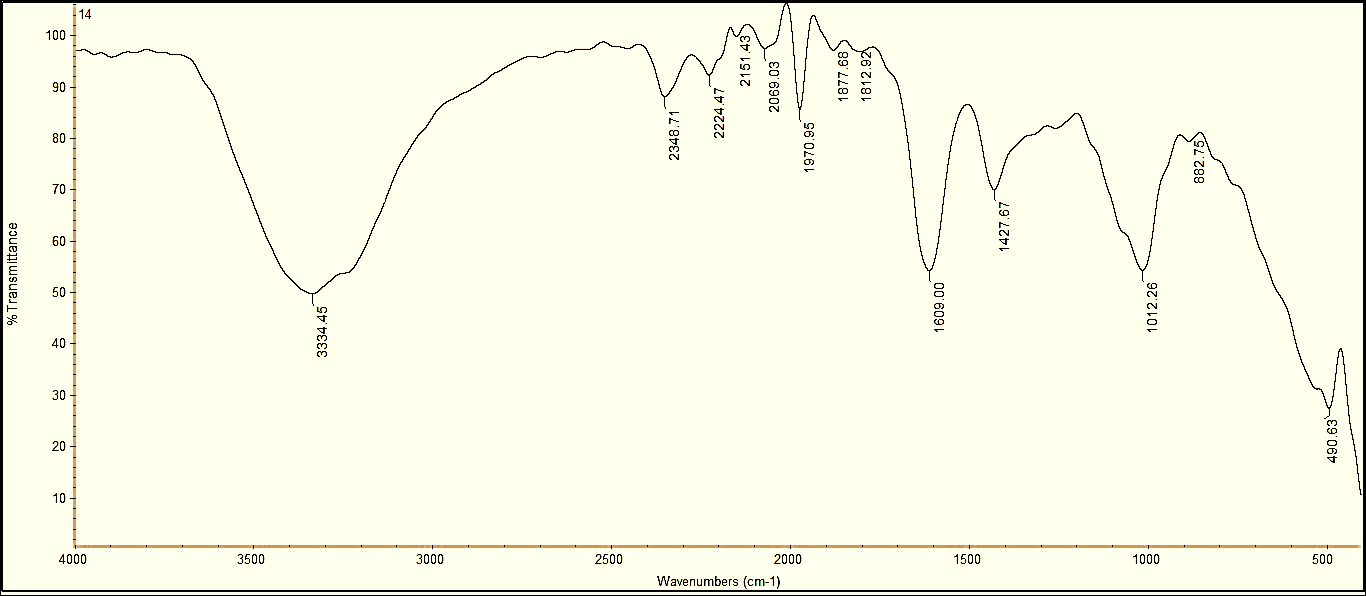


d


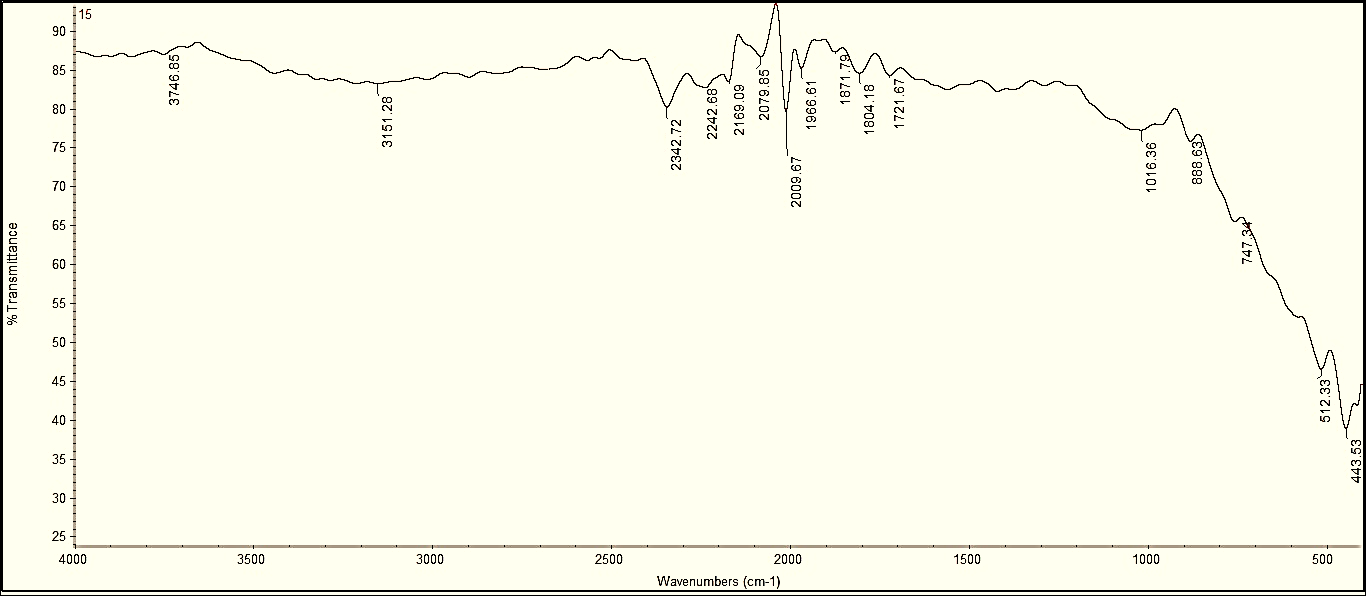


e


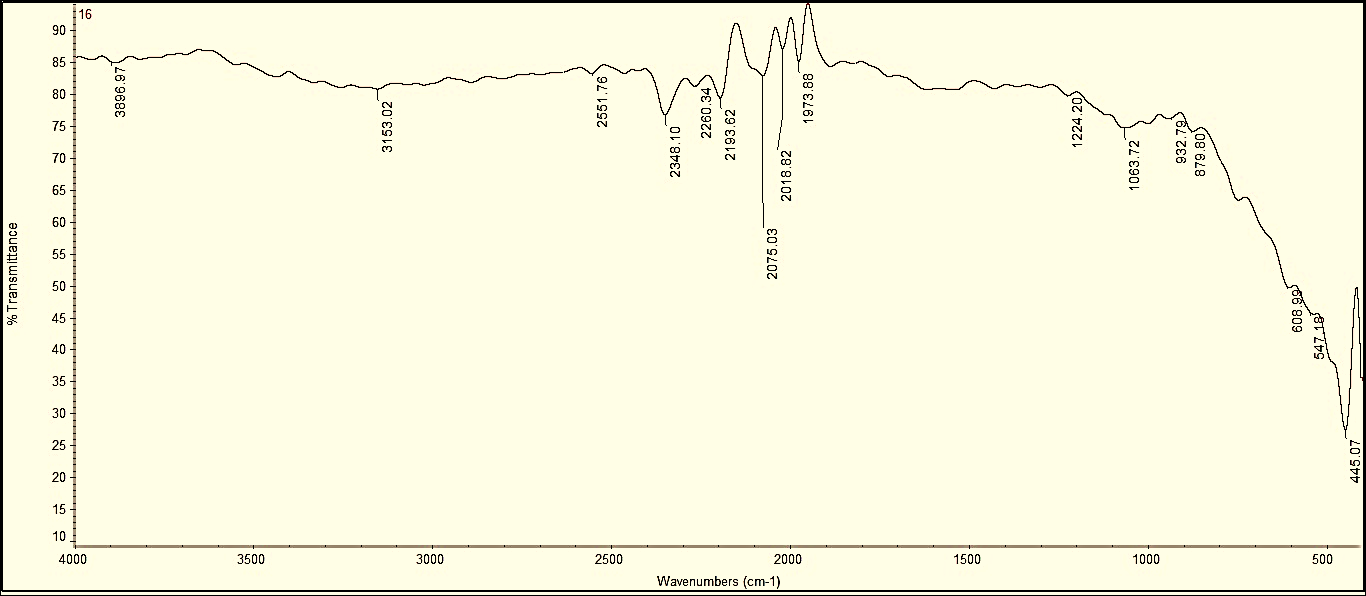


f

Fig.S2. FTIR spectra of (a)Cs; (b) GO @Cs‑GLA-TiO_2_ composite; (c) TiO_2_; (d) GO @Cs‑GLA-TiO_2_ composite after dye (e ) MO and(f) Cr(VI).

a

**Intensity(a.u.)**

**2**θ **(degree)**

**Intensity(a.u.)**

b

**2**θ **(degree)**

**Intensity(a.u.)**

c

**2**θ **(degree)**

d

**Intensity(a.u.)**

**2**θ **(degree)**

Fig. S3. (a-b-c-d) the XRD images of GO, TiO_2_,CS, and GO @Cs‑GLA-TiO_2_ composite.


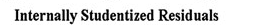

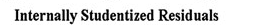

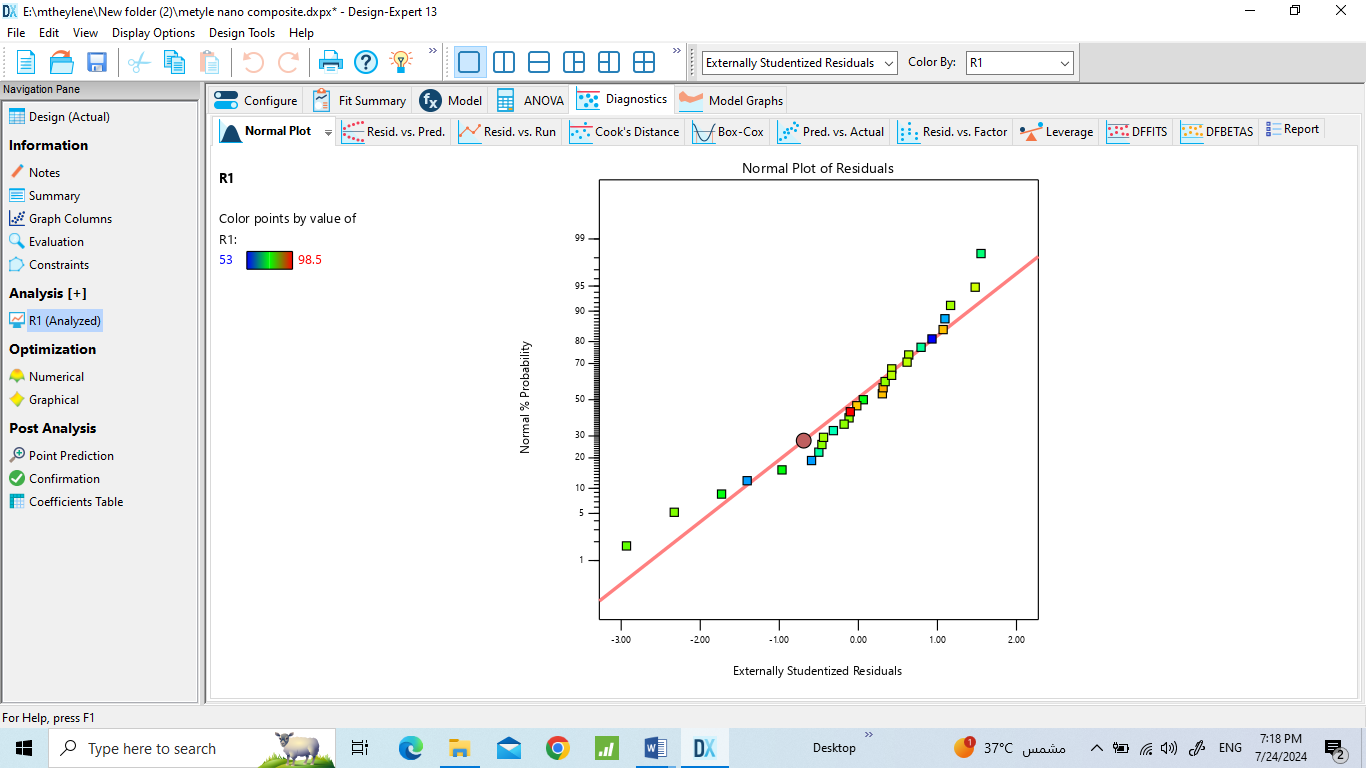


b

a


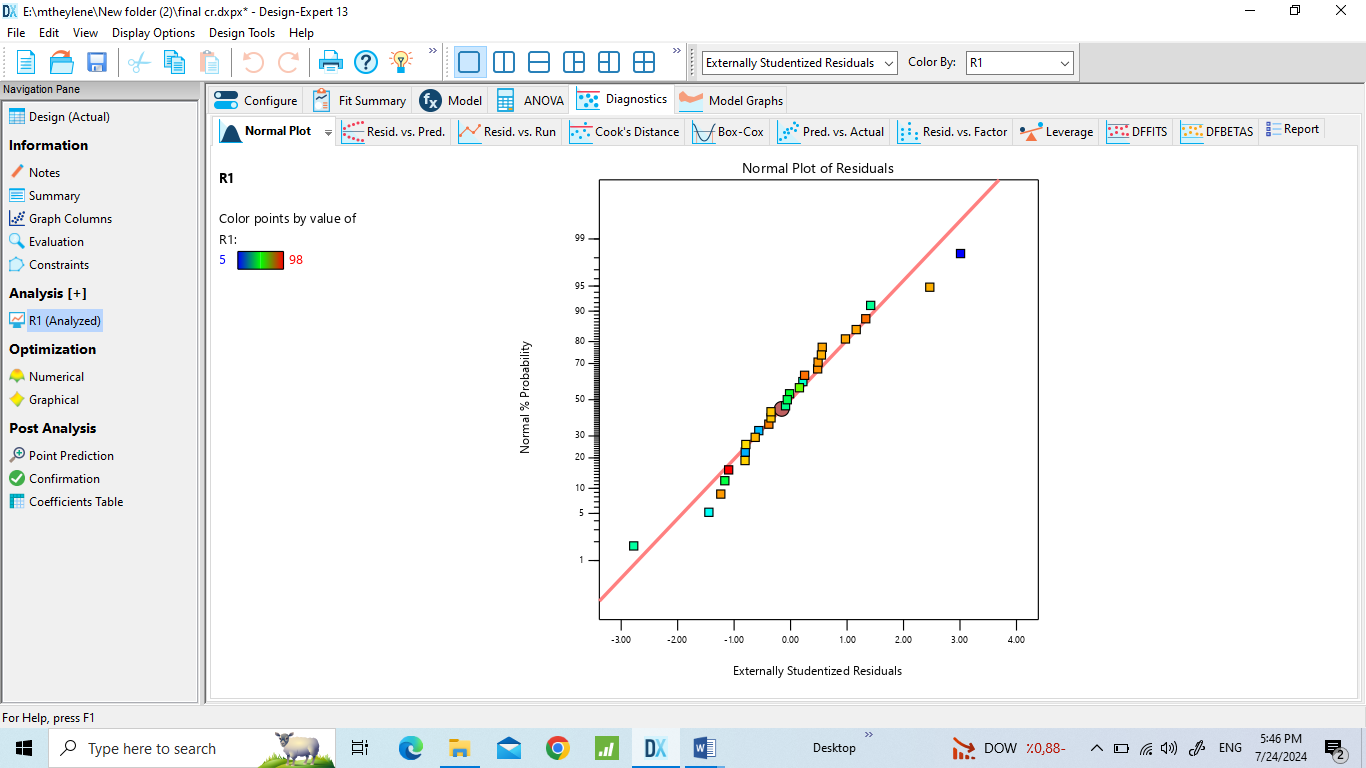


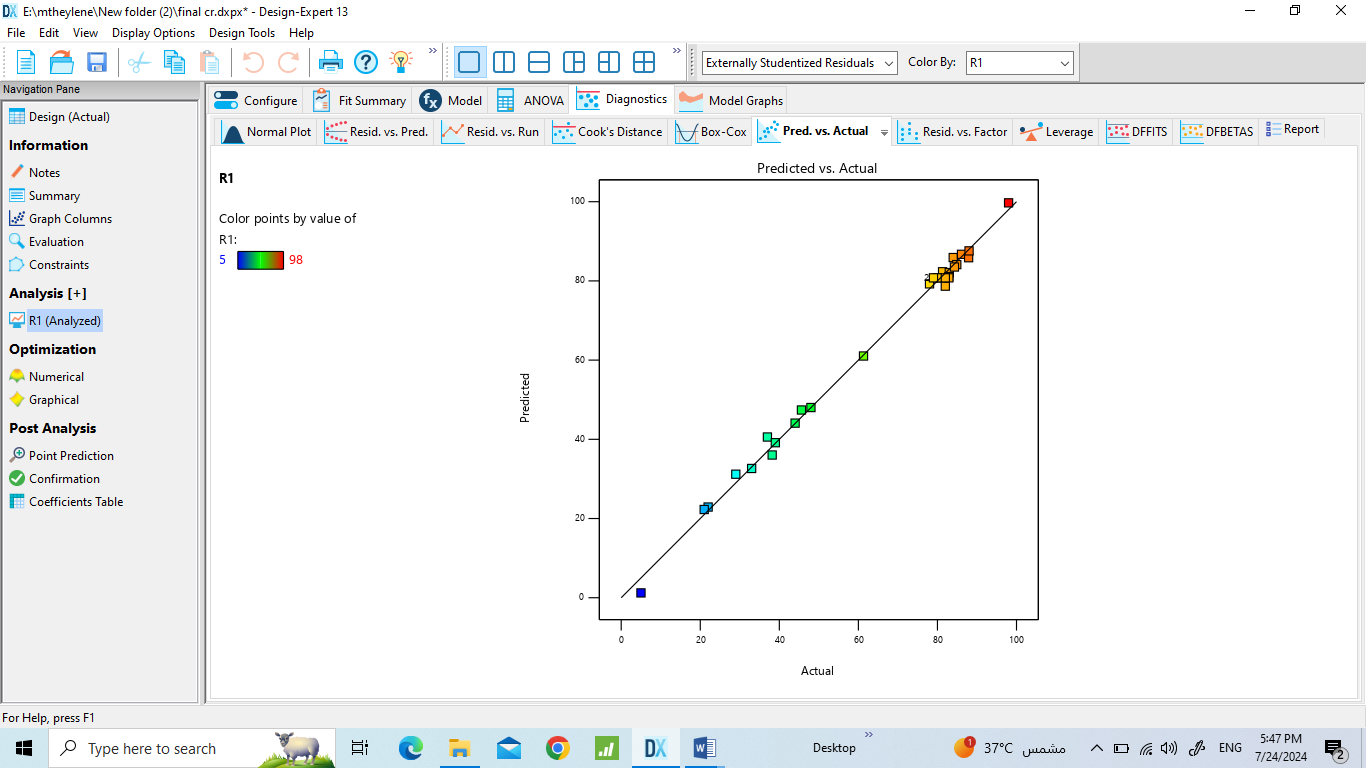

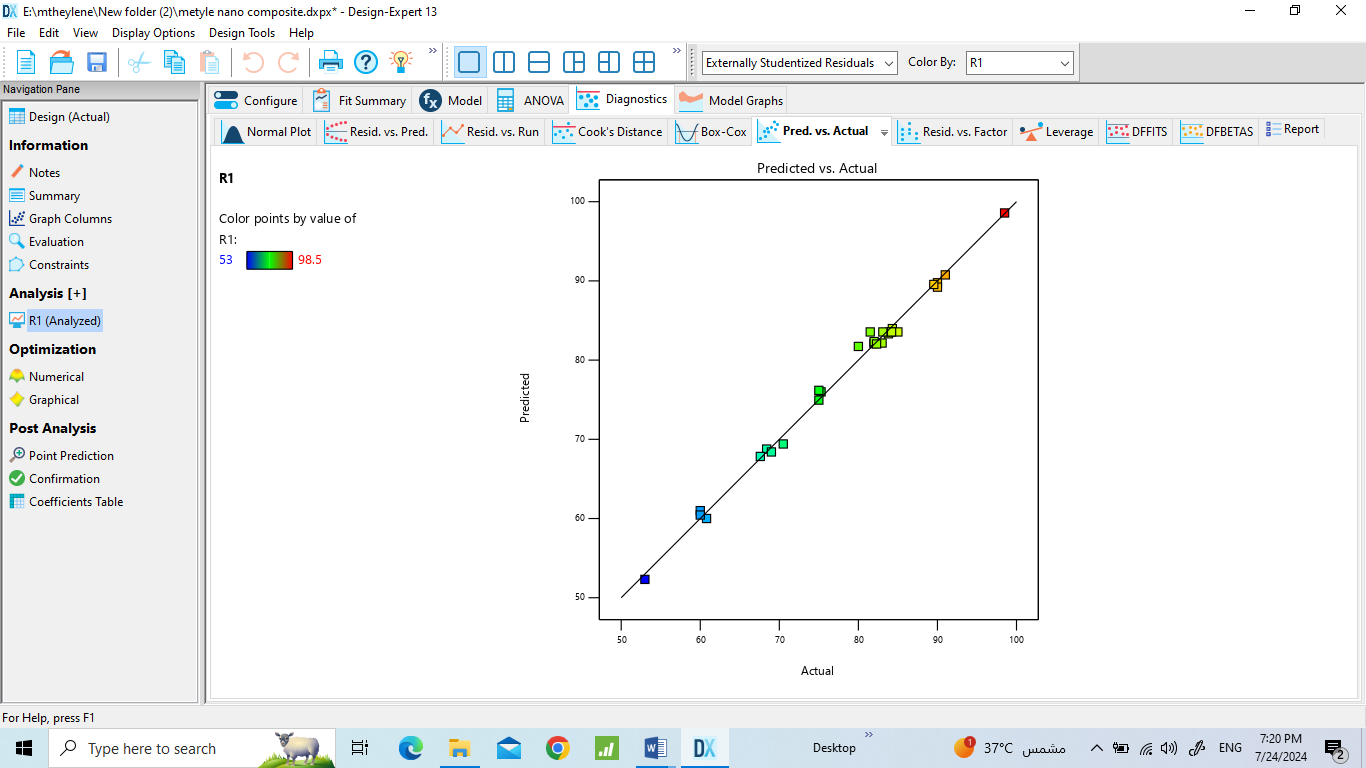


d

c


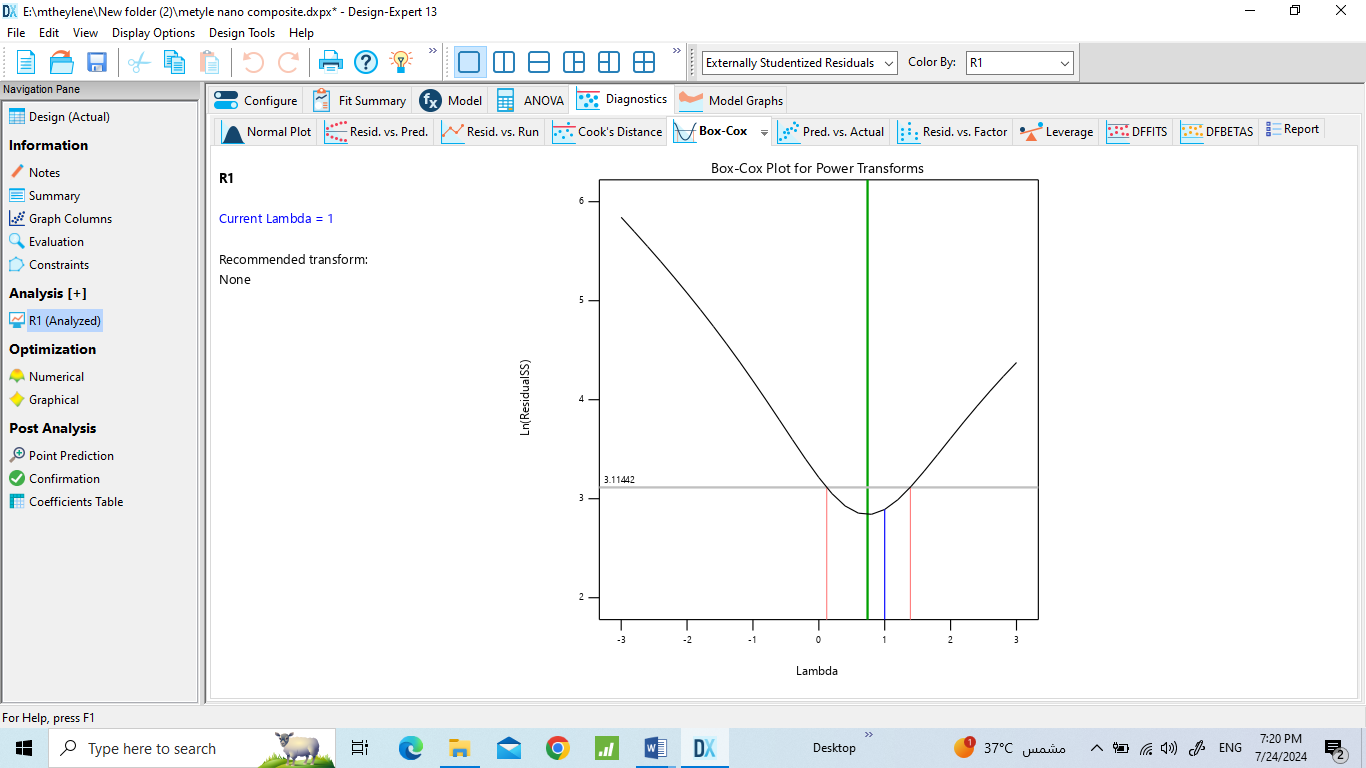

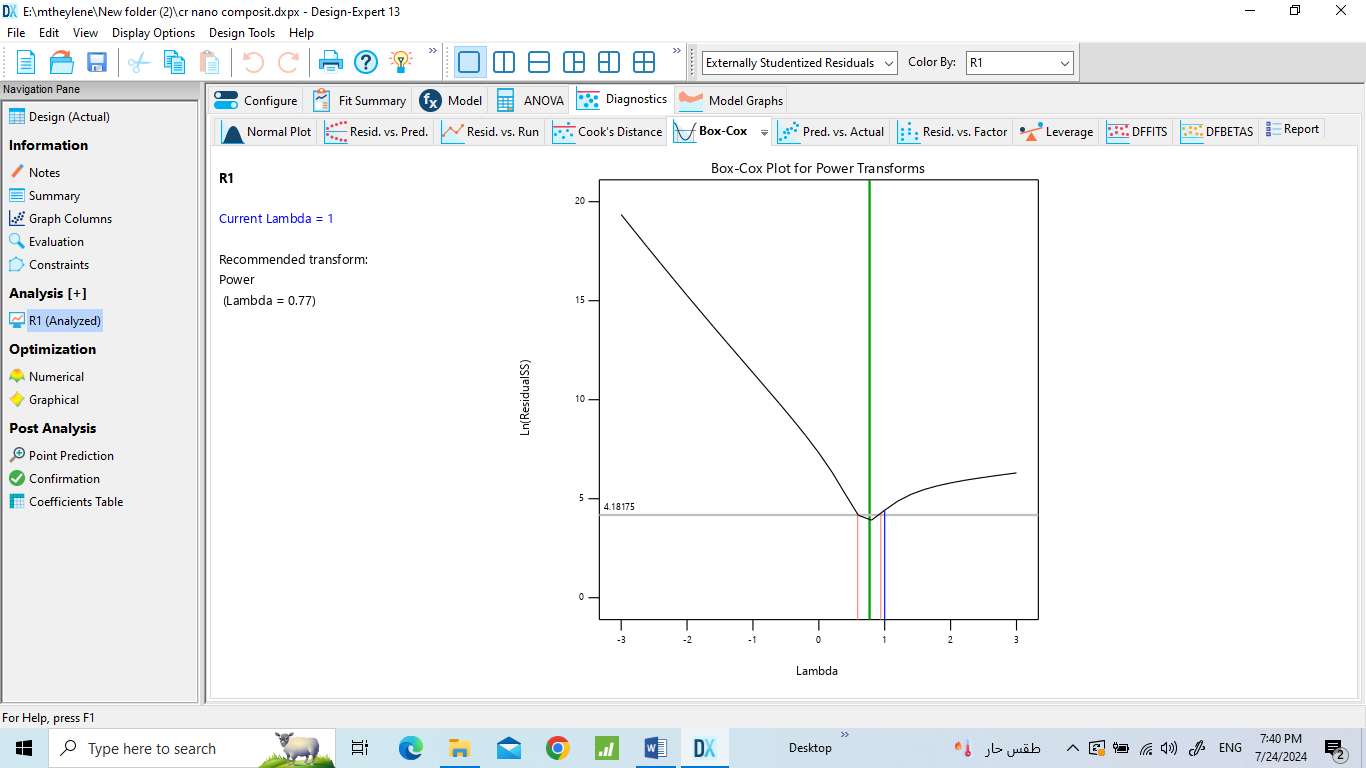


c

e

f

Fig.S4(a)The actual vs predicted removal percentage, (b) The normal probability vs the plot of the studentized residuals, (c),(d) The cook’s distance for each of the experimental runs, Box–Cox plot (e,f) each of the variables for the removal of MO and Cr(VI) adsorption on GO@Cs--GLA-TiO_2_.


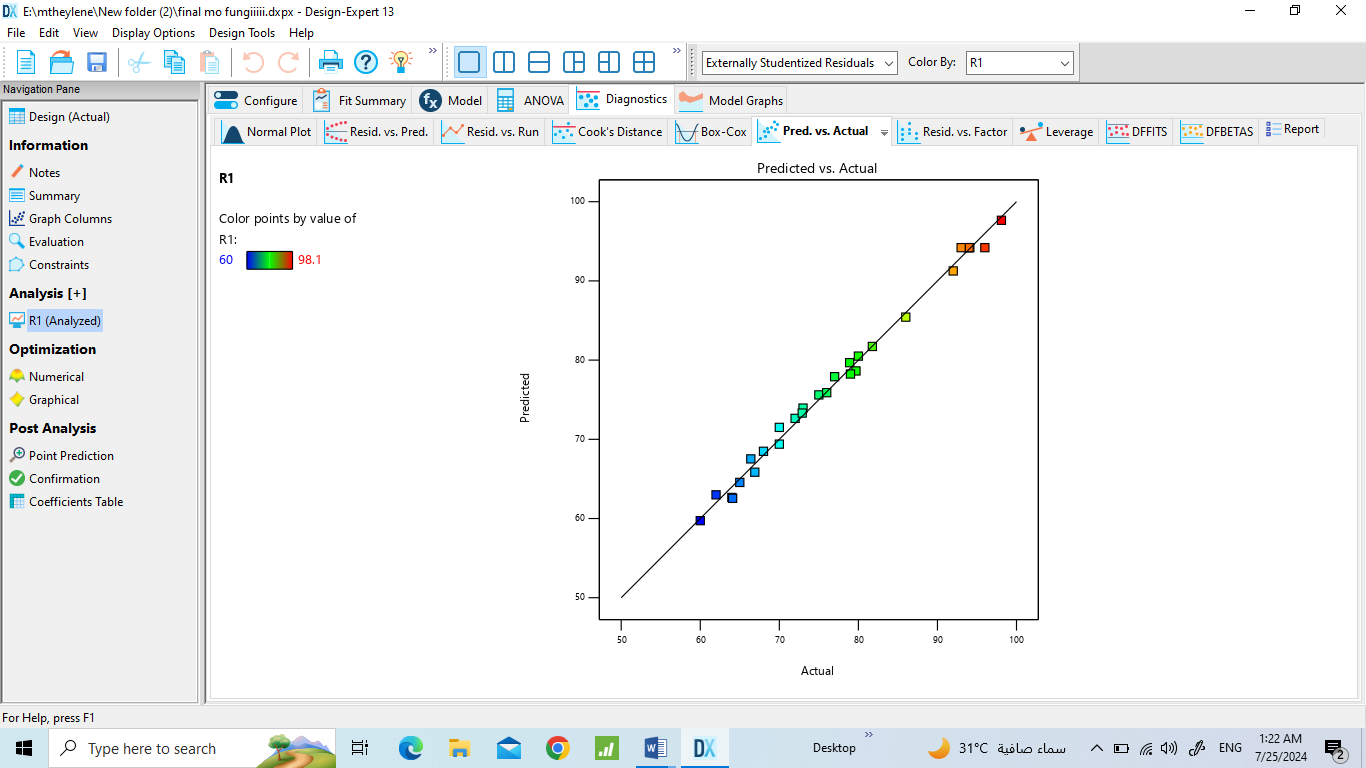

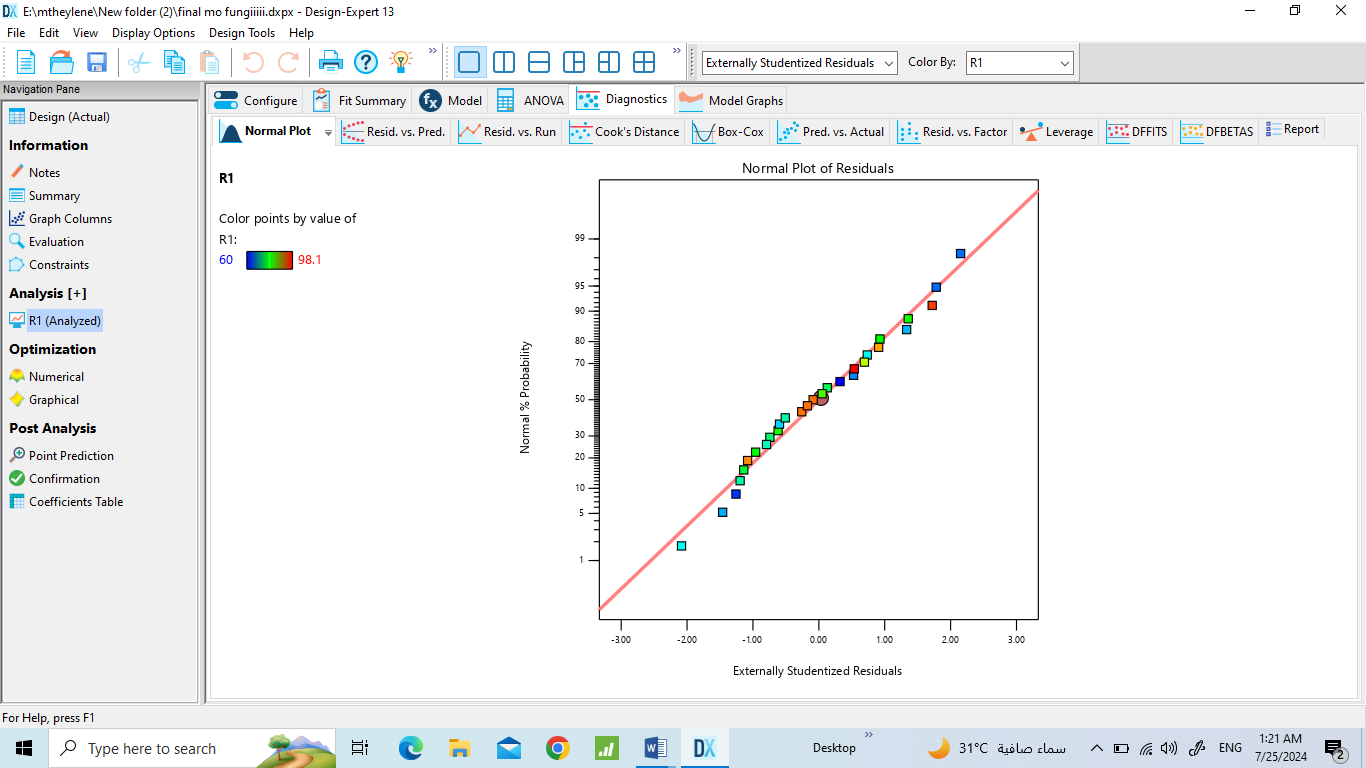

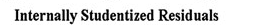


b

a


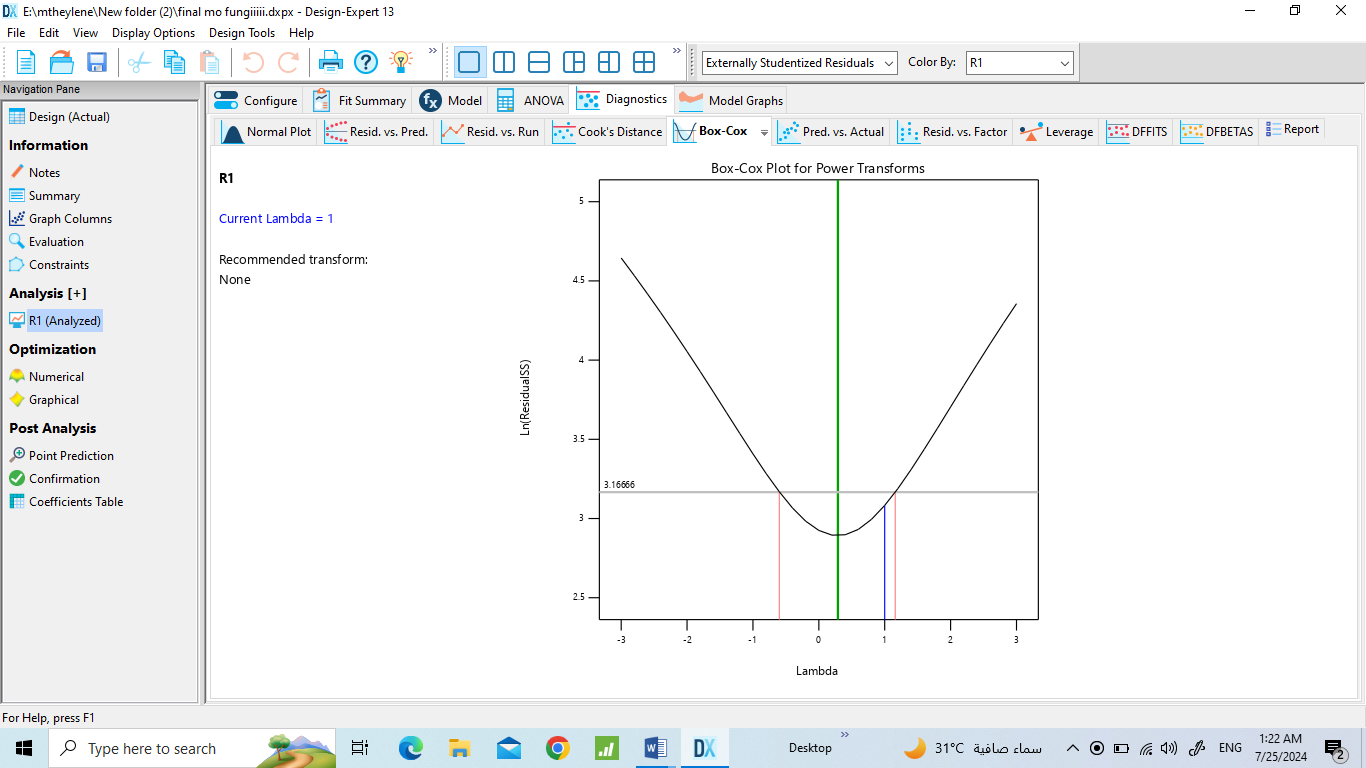

c

Fig.S5 (a)The actual vs predicted removal percentage, (b) The normal probability vs the plot of the studentized residuals, (c),(d) The cook’s distance for each of the experimental runs, each of the variables for the removal of MO adsorption on fungal@Cs-GLA-GO.


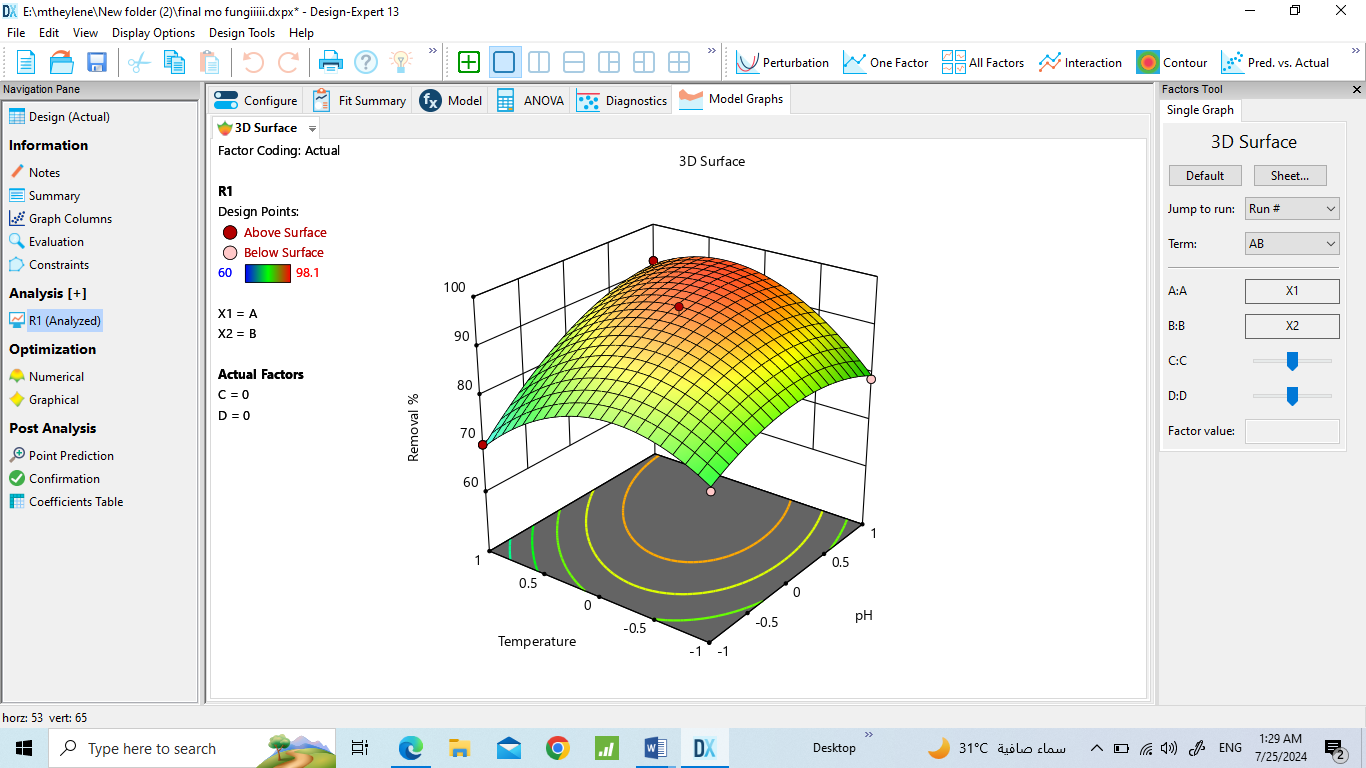


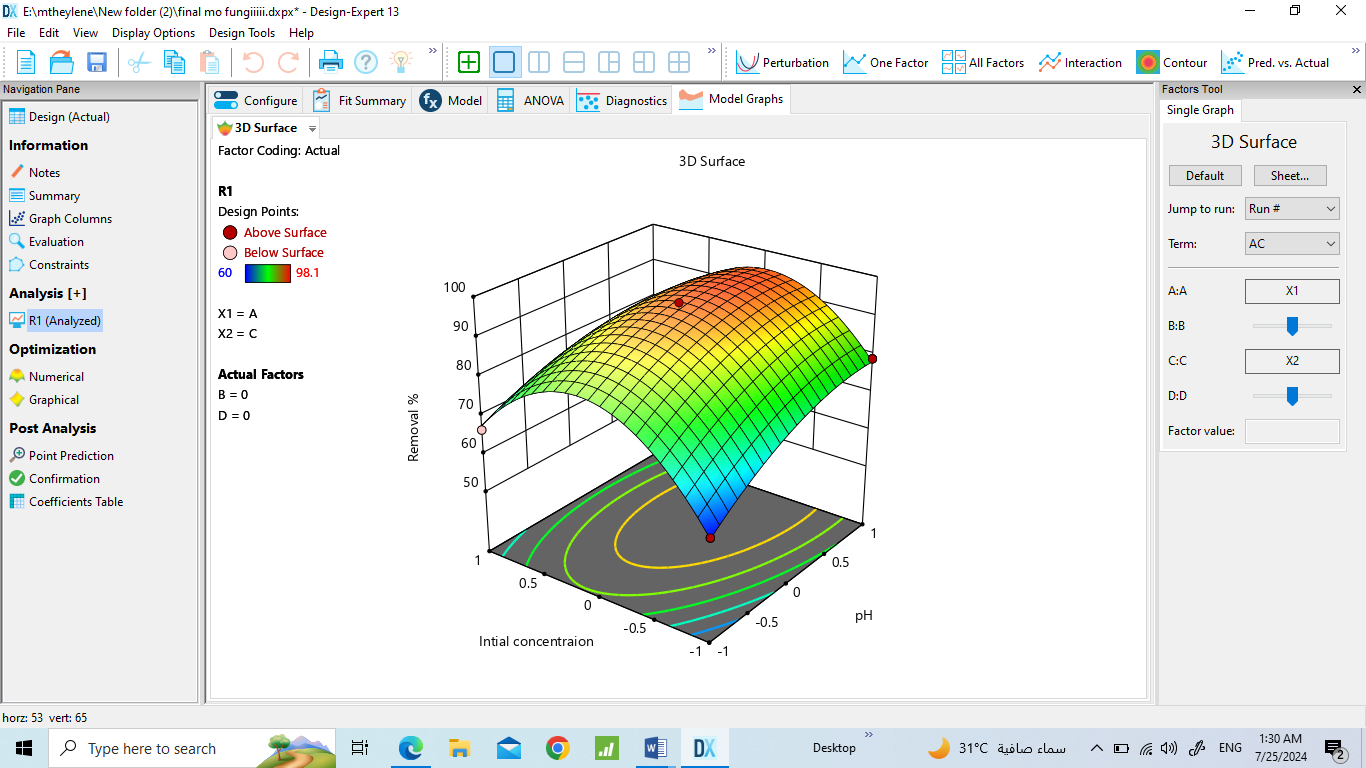


**Removal %**

**Removal %**

b

a

**Contact Time**

**Temperature**

**pH**

**Contact Time**


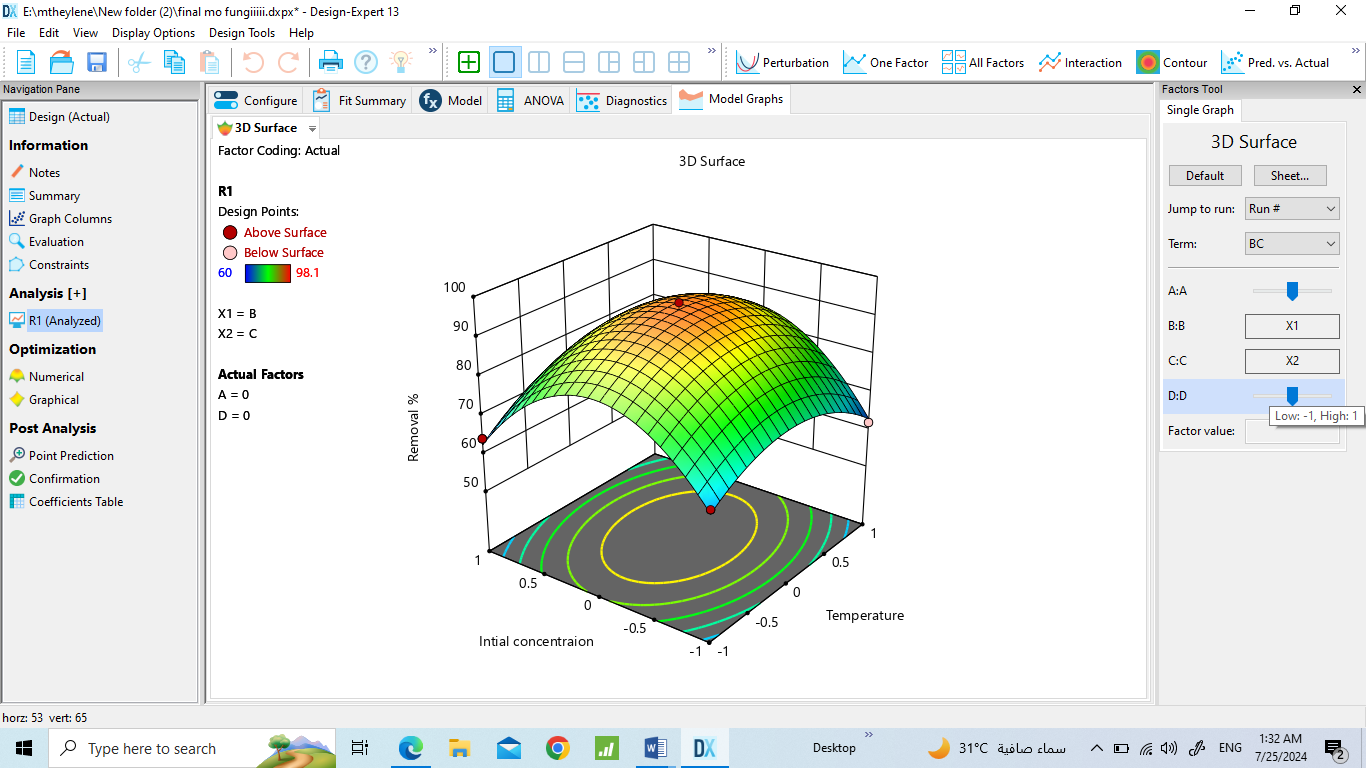

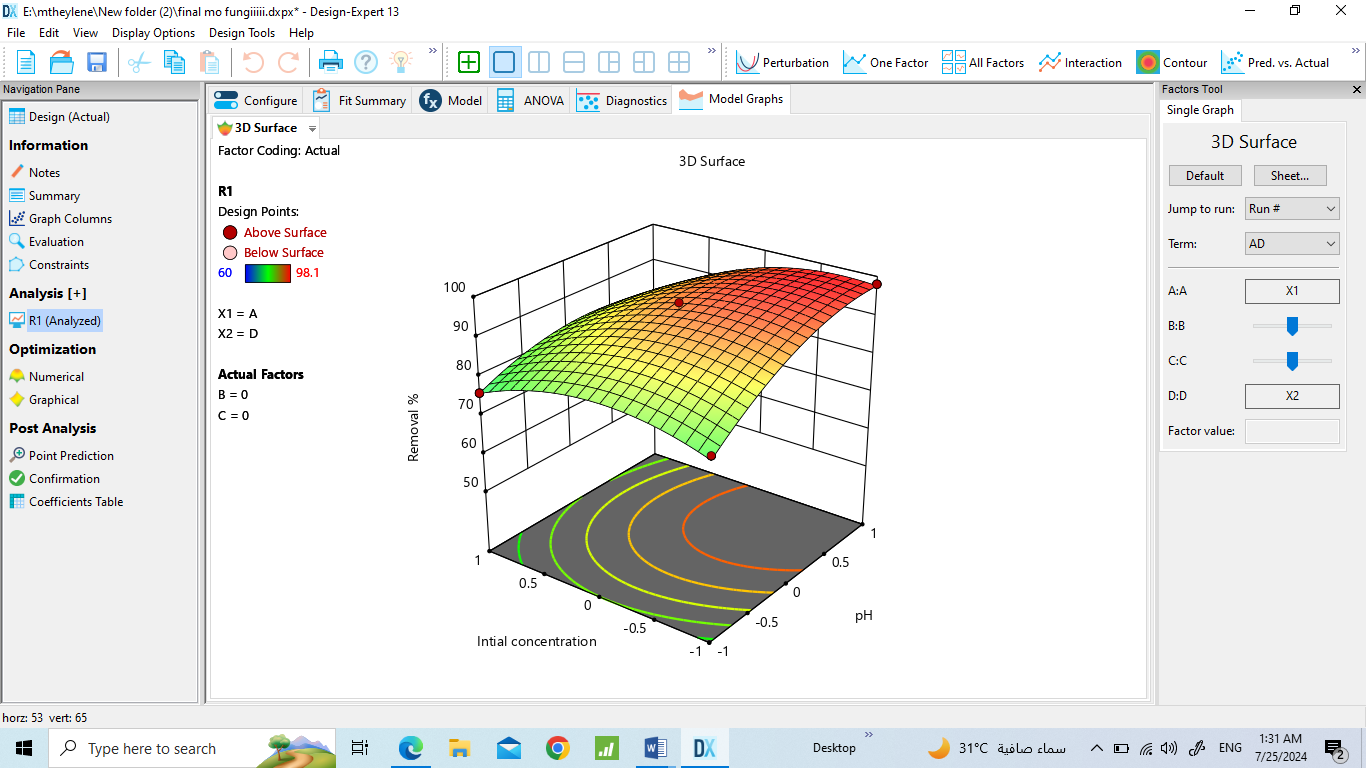


**Removal %**

**Removal %**

d

c

**Temperature**

**Initial concentration**

**pH**

**Contact Time**


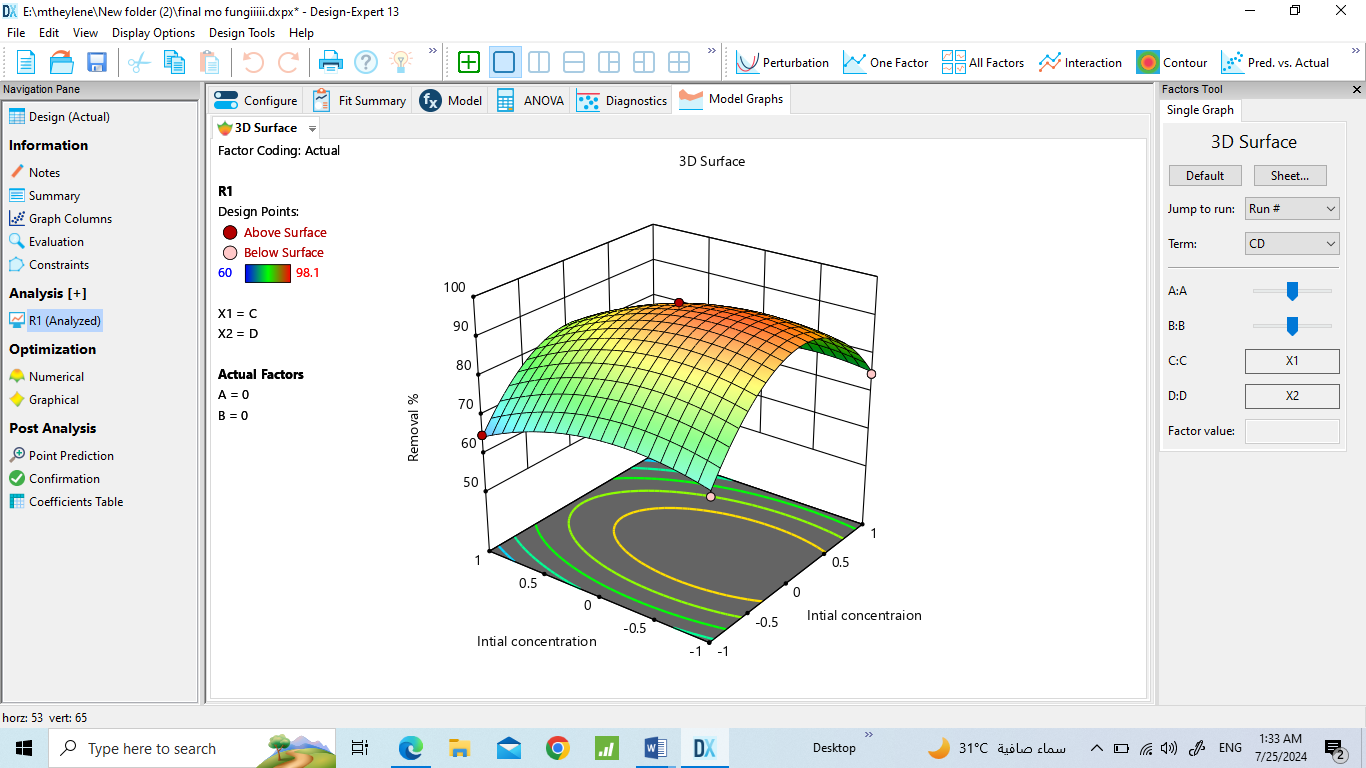


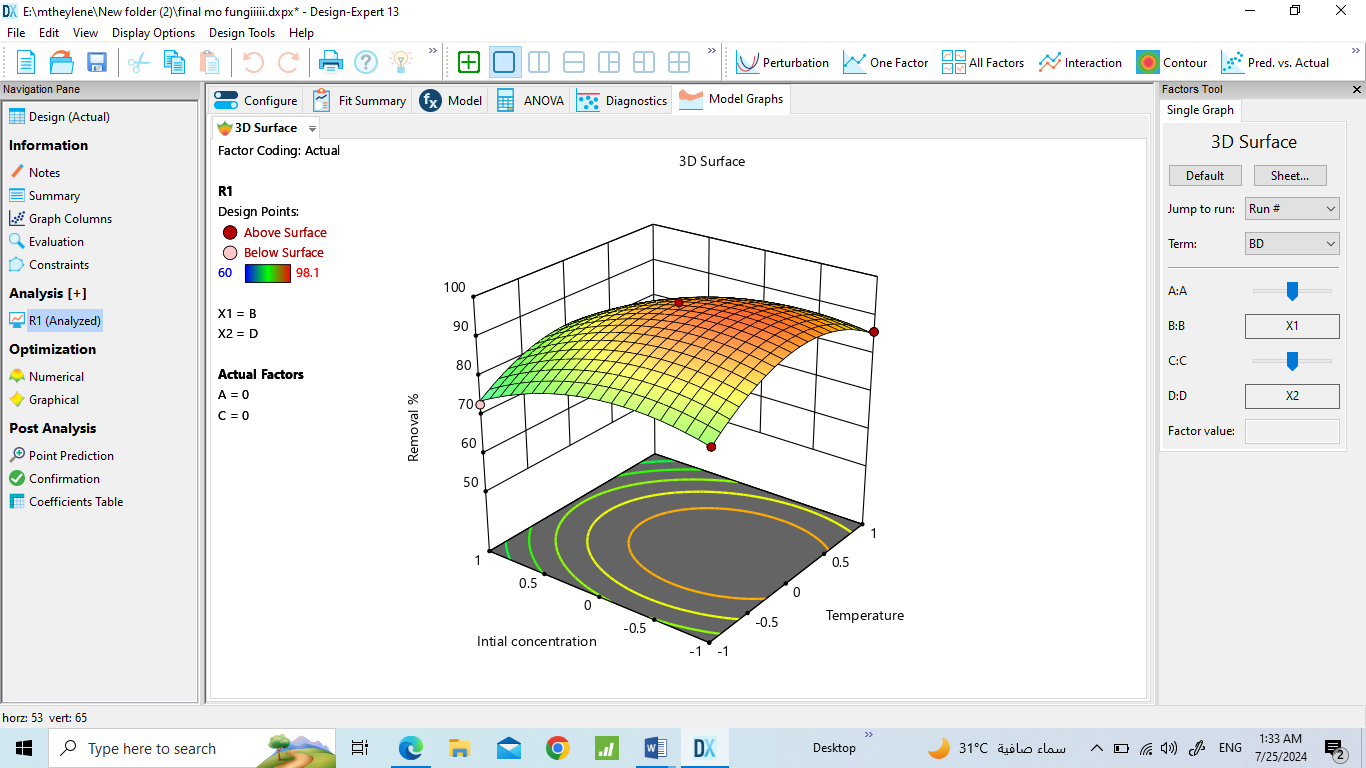


**Removal %**

**Removal %**

f

**Temperature**

e

**Initial concentration**

**Initial concentration**

**pH**


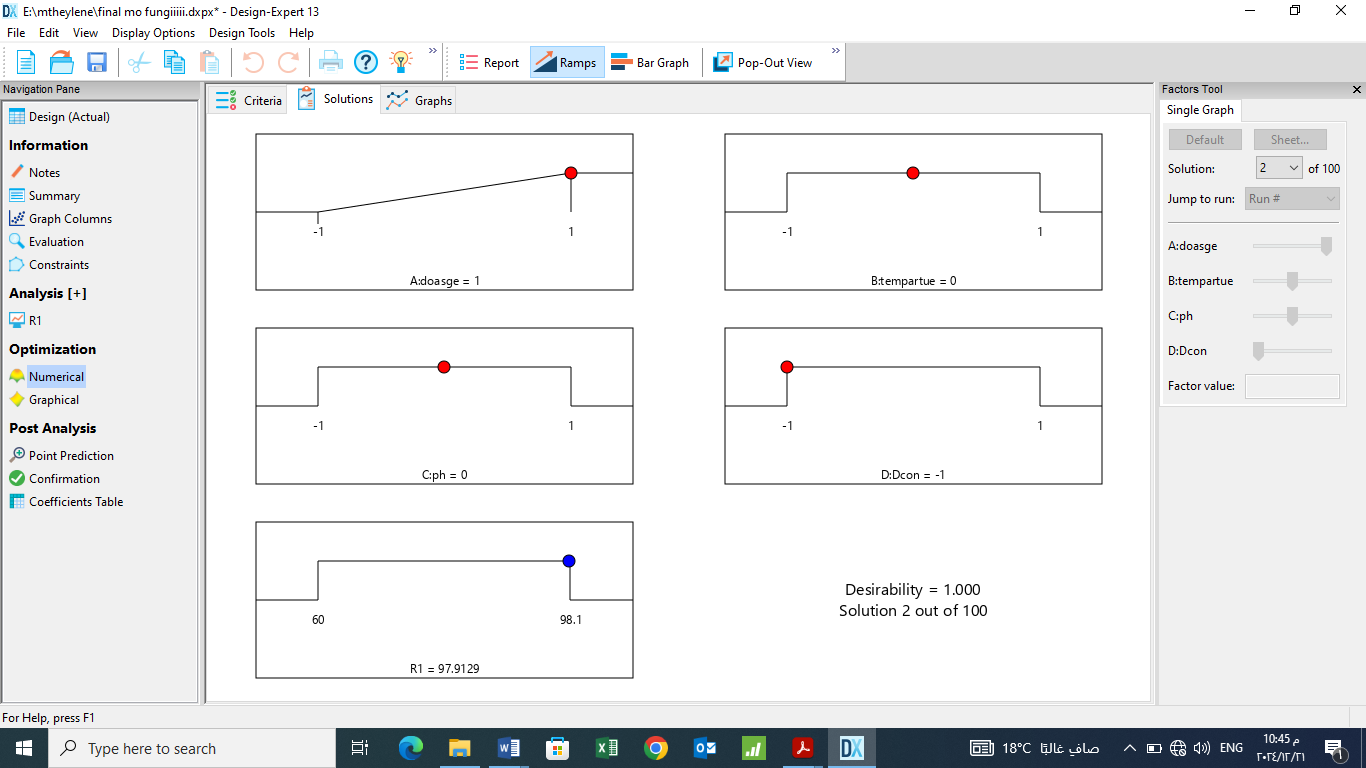


Fig. S6 Response surface graph for the removal of MO (%) by biosorbent with interactions between(a) temperature and dosage, (b) pH and dosage, and (c) initial concentration of MO (d) pH and temperature, and(e) initial concentration of MO and temperature, and (fl) pH and initial concentration of MO, and (m) desirability ramps for numerical optimization of four independent variable.


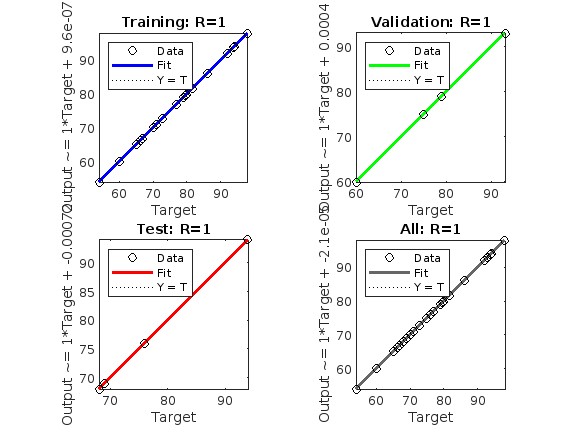


a


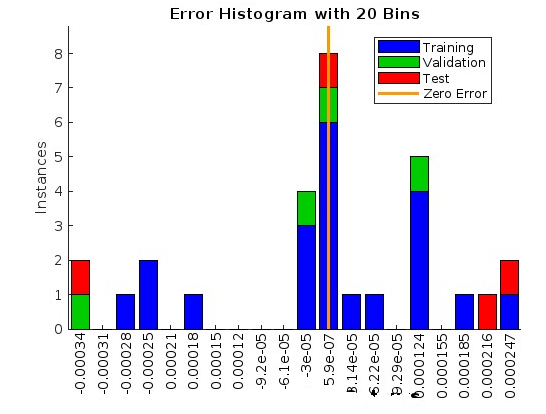


b


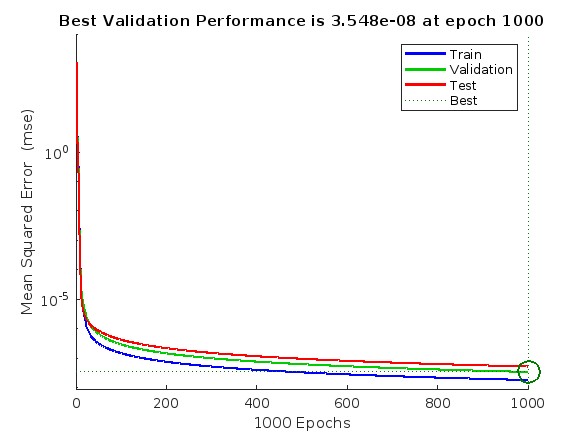


c


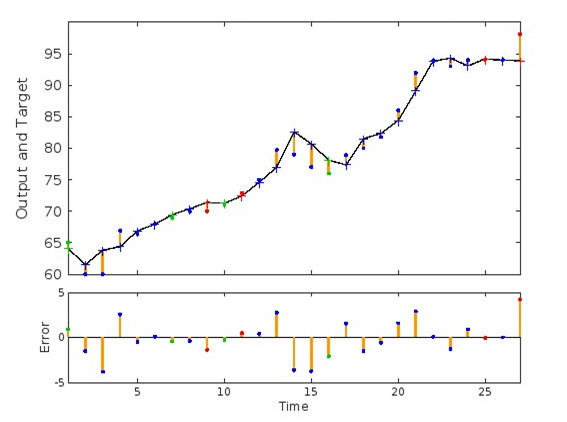


d

Fig. S7. RSM-ANN model: (a) Linear regression for the ANN-MO model training, verification, testing and combined input data sets, (b) error histogram, and (c) MSE plot for training, validation, testing, and all data and(d) The percentage of the prediction error of the MO-ANN model.

a

b

Fig. S8 Time of the lag phase (t0) versus the initial MO concentration (a), and (b) (S_0_) maximum specific growth rate μm versus the time of the lag phase t_0_ for S_0_ below

a

b

c

d

e

Fig. S9 Experimental and predicted speciﬁc substrate consumption rates at diﬀerent MO concentrations due to diﬀerent models.

Table S1 Coded levels and design range of variables.

| Factor | Code | -1 | 0 | 1 |
| --- | --- | --- | --- | --- |
| Adsorbent dose(g) | A | 0.4 | 0.6 | 0.8 |
| Contact time(min) | B | 40 | 60 | 80 |
| pH | C | 2 | 3 | 4 |
| Initial concentration (MO/Cr(VI)) (mgL^-1^) | D | 10 | 15 | 20 |

Table S2. Characterization methods and analyzed properties

| Method | Properties to be Analyzed |
| --- | --- |
| SEM Particle shape, size and morphology.  EDX | Energy-dispersive X-ray spectroscopy (EDX) was performed for the semi-quantitative test of the prepared catalysts using scanning electron microscope coupled with EDX unit (Quanta FEG250, FEI Company |
| XRD Extent of Crystallization of the sample. | X-ray diffraction (XRD) analysis was performed on X-ray diffractometer (PANalytical X’Pert Pro, United Kingdom. |
| FT-IR | The Fourier- transformed infrared (FTIR) spectra of the synthesized catalysts were obtained using Bruker-VERTEX 80V to identify the surface functional groups at ambient conditions within the wavelength range from 400 to 4000 cm^−1^. |

| Kinetic models | Linear expression | Plot | Parameters |
| --- | --- | --- | --- |
| Pseudo-first order | ln(q_e_ − q_t_) =lnq_e_ − k_1p_t eq.4 | ln(q_e_ − q_t_) vs.t | q_e_ = exp(intercept),  k_1p_ = −(slope) |
| Pseudo-second  Order | t/q_t_ = 1/k_2_pq_e_^2^ + t/qe  eq.5 | t/q_t_ vs. t | qe= slope^−1^,  k_2p_ = (slope^2^)/intercept |
| Intraparticle diffusion | q_t_ = K_p_t^1∕2^ eq.6 | q_t_  vs t^1/2^ | k_iP_ = slop  C =intercept |
| Elovich | q_t_=1βln(αβ) +1βln(t)eq.(7) | q_t_  vs t |  |

Table S3 Kinetic models and their linearized expressions in the adsorption of MO/Cr(VI)by GO@Cs-GLA-TiO_2_.

Table S4 Isotherm models and their linearized expressions in the adsorption of MO and Cr(VI) onto GO@Cs-GLA-TiO_2_ adsorbent.

| Isotherms | Equations | Linear expression | Description | Plot | Parameters |
| --- | --- | --- | --- | --- | --- |
| Langmuir | q_e_=(q_m_K_L_C_e_)/(1 +K_L_C_e_) eq.3  $\mathbf{R}_{\mathbf{L}}\mathbf{=}\frac{\mathbf{1}}{\mathbf{1+}\mathbf{K}_{\mathbf{L}}\mathbf{C}_{\boldsymbol{^{\circ}}}}$ eq.(8) | C_e_/q_e_ = (1/KLq_m_) + (C_e_/q_m_) | qmax (mg/  g) = maximum adsorption capacity.  K_L_ (L/mg) = constant of Langmuir (free energy of adsorption) | (C_e_/q_e_) vs. C_e_ | q_m_= (slope)^−1^  K_L_= slope/intercept |
| Freundlich | q_e_=K_F_(C_e_)^1/n^  eq.9 | $lnq_{e}=lnK_{F}+\frac{1}{n}lnC_{e}$ | KF (mL/g) = constants of Freundlich (the adsorption volume of the adsorbent)  1/n = constants (intensity of the analytes’ sorption) | ln q_e_ vs. ln C_e_ | K_F_= exp(intercept), n = (slope)^−1^ |
| Temkin | q_e_=q_m_ ln(KTC_e_) eq. 10 | q_e_=q_m_ ln KT + q_m_ ln C_e_ | ΔQ = variation of adsorption energy (ΔH)  K_0_ = constant of Temkin. T = temperature (K)  R (8.314 J/mol) K = universal gas constant. | q_e_ vs. ln C_e_ | q_m_= slope  KT= exp(intercept/slope |
|  | qe =KRp Ce/ 1+αRP (Ce ^β^ ) | ln KRP/ qe ^−1^ = βln (Ce)+ln( α RP) |  |  |  |

| Factor | Code | -1 | 0 | 1 |
| --- | --- | --- | --- | --- |
| pH | A | 5 | 7 | 9 |
| Temperature(^O^C) | B | 20 | 30 | 40 |
| Contact time(day) | C | 3 | 6 | 9 |
| Initial concentration (mgL^-1^) | D | 10 | 30 | 50 |

Table S5(a) Coded levels and design range of variables.

Table S5 (b)The ANOVA results for the response surface model for the removal of MO dye.

| Source | Sum of Squares | df | Mean Square | F-value | p-value |  |
| --- | --- | --- | --- | --- | --- | --- |
| Model | 3701.07 | 14 | 264.36 | 169.60 | < 0.0001 | significant |
| A-A | 418.90 | 1 | 418.90 | 268.74 | < 0.0001 |  |
| B-B | 7.10 | 1 | 7.10 | 4.55 | 0.0510 |  |
| C-C | 3.63 | 1 | 3.63 | 2.33 | 0.1493 |  |
| D-D | 296.71 | 1 | 296.71 | 190.35 | < 0.0001 |  |
| AB | 101.00 | 1 | 101.00 | 64.80 | < 0.0001 |  |
| AC | 44.89 | 1 | 44.89 | 28.80 | < 0.0001 |  |
| AD | 51.84 | 1 | 51.84 | 33.26 | < 0.0001 |  |
| BC | 19.36 | 1 | 19.36 | 12.42 | 0.0034 |  |
| BD | 4.69 | 1 | 4.69 | 3.01 | 0.1049 |  |
| CD | 9.00 | 1 | 9.00 | 5.77 | 0.0307 |  |
| A² | 164.52 | 1 | 164.52 | 105.55 | < 0.0001 |  |
| B² | 598.11 | 1 | 598.11 | 383.70 | < 0.0001 |  |
| C² | 2497.89 | 1 | 2497.89 | 1602.46 | < 0.0001 |  |
| D² | 232.74 | 1 | 232.74 | 149.31 | < 0.0001 |  |
| Residual | 21.82 | 14 | 1.56 |  |  |  |
| Lack of Fit | 17.00 | 10 | 1.70 | 1.41 | 0.3958 | not significant |
| Pure Error | 4.82 | 4 | 1.21 |  |  |  |
| Cor Total | 3722.90 | 28 |  |  |  |  |

Table S5c Box–Behnken design-based experimental conditions and results for MO adsorption activity of composites fungal@Cs-GLA-GO_._

|  | Factor 1 | Factor 2 | Factor 3 | Factor 4 | Response 1 |
| --- | --- | --- | --- | --- | --- |
| Run | A:A | B:B | C:C | D:D | R1 |
|  |  |  |  |  |  |
| 1 | 0 | 0 | 1 | 1 | 64 |
| 2 | 0 | -1 | 1 | 0 | 64.1 |
| 3 | 0 | 0 | -1 | 1 | 65 |
| 4 | 0 | 1 | -1 | 0 | 62 |
| 5 | -1 | 0 | -1 | 0 | 60 |
| 6 | 0 | -1 | -1 | 0 | 66.9 |
| 7 | -1 | 0 | 1 | 0 | 66.4 |
| 8 | 0 | 1 | 1 | 0 | 68 |
| 9 | 1 | 0 | 1 | 0 | 72 |
| 10 | -1 | 1 | 0 | 0 | 70 |
| 11 | 0 | 0 | -1 | -1 | 70 |
| 12 | 0 | -1 | 0 | 1 | 73 |
| 13 | 0 | 1 | 0 | 1 | 72.9 |
| 14 | 0 | 0 | 1 | -1 | 75 |
| 15 | -1 | 0 | 0 | -1 | 79.7 |
| 16 | 1 | 0 | -1 | 0 | 79 |
| 17 | -1 | -1 | 0 | 0 | 77 |
| 18 | -1 | 0 | 0 | 1 | 76 |
| 19 | 1 | -1 | 0 | 0 | 78.9 |
| 20 | 1 | 0 | 0 | 1 | 80 |
| 21 | 0 | -1 | 0 | -1 | 81.77 |
| 22 | 0 | 1 | 0 | -1 | 86 |
| 23 | 1 | 1 | 0 | 0 | 92 |
| 24 | 0 | 0 | 0 | 0 | 93.9 |
| 25 | 0 | 0 | 0 | 0 | 93 |
| 26 | 0 | 0 | 0 | 0 | 96 |
| 27 | 0 | 0 | 0 | 0 | 94.1 |
| 28 | 0 | 0 | 0 | 0 | 94 |
| 29 | 1 | 0 | 0 | -1 | 98.1 |

Table S6: Various kinetic models for the effect of substrate on growth rate

| Models | Equation | μmax (hr^–1^) | Ks (mg/L) | Ki | RMSE | AICc | R^2^ |
| --- | --- | --- | --- | --- | --- | --- | --- |
| Haldane | $\mu=\frac{\mu maxS}{(S+KS+\frac{S^2}{Ki})}$ eq.(16) | 0.5 | 30 | 100 | 0.009 | -30.9 | 0.95 |
| Han and Levenspiel | $\mu=\frac{\mu maxS\left[ 1-\left( \frac{S}{Sm} \right) \right]n}{\left( S+KS- \right)\left[ 1-\left( \frac{S}{Sm} \right) \right]m}$ eq.(17) | 1 | 30 |  | 0.022 | -29.02 | 0.91 |
| Luong | $\mu=\frac{\mu maxS\left[ 1-\left( \frac{S}{Sm} \right) \right]n}{(S+KS)}$ eq.(18) | 0.7 | 15 |  | 0.05 | -26.4 | 0.90 |
| Aiba | $\mu=\frac{\mu maxS exp(- S/Ki)}{(S + KS)}$ eq.(19) | 1.3 | 10 | 40 | 0.04 | -20.7 | 0.85 |
| Monod | $\mu max=\frac{S}{KS}$ eq.(20) | 0.9 | 5 |  | 0.23 | -14.2 | 0.53 |

**Qmax:** Maximal Reduction Rate (h^-1^)

**ks** :Half saturation constant for maximal reduction (mg/dm^3^)

**RMS:** Residual mean square

Table S7 Comparative study for different biomass-based adsorbents applied for methyl orange adsorption.

| Adsorbents | Removal efficiency/adsorption capacity MO/Cr(VI) | References |
| --- | --- | --- |
| Powdered Rumex abyssinicus | 98.5 % (3.7mg/g) (MO) | (Abewaa et al., 2023) |
| CO_3_O_4_ nanoparticles | 15% (46.08 mg/g) MO | (Baig et al., 2020) |
| CuO-ZnO composite | 78% 63.25 mg/g MO | (Najafidoust et al., 2024) |
| Graphene oxide/ZnO | 95% (Cr(VI) (125mg/g) | (Asl et al., 2013) |
| zero-valent iron (S-nZVI) supported on hydrogel | 87.7%(Cr(VI) | (Zhang et al., 2019) |
| PANI@WH composites | 98.5% (31 mg/g)(Cr(VI) | (Kumari et al., 2022) |
| Nnano-magnetic Enteromorpha prolifera hydrogel | 73% (231 mg/g) of MO/ Cr(VI)  54.2 mg/g. 25% | (Yang et al., 2023) |
| GO/ZnO nanocomposites | MO (88%) | (Moradi et al., 2022) |
| Biopolymer chitosan | 78.12 mg g-1 (80%)MO | (Khalil et al., 2021) |
| Jackfruit leaves powder | (78%)MO | (Dutta et al., 2022) |
| GO @Cs‑GLA-TiO_2_ | 277.7 mg/g) of MO/ Cr(VI)  33.89 mg/g | This study |

Table S8 Cost analysis of materials used for adsorption using GO @Cs‑GLA-TiO_2_

| Cost of preparing GO @Cs‑GLA-TiO_2_ for one adsorption | The raw materials used for preparing | Unit price (EGP/kg) | ($/kg) | Amount of raw material required for one adsorption(Kg) |
| --- | --- | --- | --- | --- |
|  | NaOH (99.3%) | 500 | 10 | 100X10^-3^ |
|  | GO | 1500 |  |  |
|  | Distilled water for washing | 1.08 | 0.0216 | 100X10^-3^ |
|  | GLA | 2500 | 0.8 |  |
|  | Cs | 600 | 12 |  |
|  | TiO_2_ | 5000 | 100 |  |
| Cost of electrical energy consumption during drying at 80^O^c for 4h($) |  | 3.58 | 0.286 |  |
| The estimated total cost for GO @Cs‑GLA-TiO_2_ ($)+ raw materials cost($) + cost of electrical energy($) |  |  | 0.123 |  |
